# Propolis in Oral Healthcare: Antibacterial Activity of a Composite Resin Enriched With Brazilian Red Propolis

**DOI:** 10.3389/fphar.2021.787633

**Published:** 2021-11-29

**Authors:** José Marcos dos Santos Oliveira, Théo Fortes Silveira Cavalcanti, Ingrid Ferreira Leite, Dávida Maria Ribeiro Cardoso dos Santos, Isabel Cristina Celerino de Moraes Porto, Fernanda Lima Torres de Aquino, Artur Falqueto Sonsin, Renata Matos Lamenha Lins, Rafael Pino Vitti, Johnnatan Duarte de Freitas, Emiliano de Oliveira Barreto, Samuel Teixeira de Souza, Regianne Umeko Kamiya, Ticiano Gomes do Nascimento, Josealdo Tonholo

**Affiliations:** ^1^ Postgraduate Program of Chemistry and Biotechnology, Institute of Chemistry and Biotechnology, Federal University of Alagoas, Maceió, Brazil; ^2^ Postgraduate Program in Health Research, Cesmac University Center, Maceió, Brazil; ^3^ Postgraduate Program in Materials, Center of Technology, Federal University of Alagoas, Maceió, Brazil; ^4^ Faculty of Dentistry, Federal University of Alagoas, Maceió, Brazil; ^5^ Postgraduate Program Multicenter of Biochemistry and Molecular Biology, Institute of Pharmaceutical Sciences, Federal University of Alagoas, Maceió, Brazil; ^6^ Postgraduate Program in Pharmaceutical Sciences, Institute of Pharmaceutical Sciences, Federal University of Alagoas, Maceió, Brazil; ^7^ Postgraduate Program in Health Sciences, Institute of Biological and Health Sciences, Federal University of Alagoas, Maceió, Brazil; ^8^ Postgraduate Program in Physics, Institute of Physics, Federal University of Alagoas, Maceió, Brazil; ^9^ Faculty of Dentistry, Hermínio Ometto Foundation, Araras, Brazil; ^10^ Department of Chemistry, Federal Institute of Alagoas, Maceió, Brazil

**Keywords:** propolis, antibacterial activity, *Streptococcus mutans*, dental materials, mechanical properties, fibroblasts, composite resin, direct contact test

## Abstract

The aim of this study was to obtain a Brazilian red propolis (BRP) enriched composite resin and to perform the characterization of its antibacterial activity, mechanical, and physical-chemical properties. Brazilian red propolis ethyl acetate extract (EABRP) was characterized by LC-ESI-Orbitrap-FTMS, UPLC-DAD, antibacterial activity, total flavonoids content, and radical scavenging capacity. BRP was incorporated to a commercial composite resin (RC) to obtain BRP enriched composite at 0.1, 0.15 and 0.25% (RP10, RP15 and RP25, respectively). The antibacterial activity RPs was evaluated against *Streptococcus mutans* by contact direct test and expressed by antibacterial ratio. The RPs were characterized as its cytotoxicity against 3T3 fibroblasts, flexural strength (FS), Knoop microhardness (KHN), post-cure depth (CD), degree of conversion (DC%), water sorption (Wsp), water solubility (Wsl), average roughness (Ra), and thermal analysis. Were identified 50 chemical compounds from BRP extract by LC-ESI-Orbitrap-FTMS. EABRP was bacteriostatic and bactericide at 125 and 500 μg/ml, respectively. The RP25 exhibited antibacterial ratio of 90.76% after 1 h of direct contact with *S. mutans* (*p* < 0.0001) while RC no showed significative antibacterial activity (*p* = 0.1865), both compared with cell control group. RPs and RC no showed cytotoxicity. RPs exhibited CD from 2.74 to 4.48 mm, DC% from 80.70 to 83.96%, Wsp from 17.15 to 21.67 μg/mm^3^, Wsl from 3.66 to 4.20 μg/mm^3^, Ra from 14.48 to 20.76 nm. RPs showed thermal resistance between 448–455°C. The results support that propolis can be used on development of modified composite resins that show antibacterial activity and that have compatible mechanical and physical-chemical properties to the indicate for composite resins.

## Introduction

Propolis is a natural, nontoxic, raw material collected by *Apis mellifera* bee from plants sources as exudates, tree barks and leaf buds from various plant sources (Koo et al., 2002; da silva Barboza et al., 2021). Diverse types of propolis are known around the world and each type differs in its chemical composition, this variation occurs in function of its botanic origin, a local main plant source used by bee for propolis obtention (Teixeira et al., 2005; Daugsch et al., 2008; Ristivojević et al., 2015). The botanical origin of red propolis collected in northeastern Brazil is the *Dalbergia ecastaphyllum* (L.) Taub*.* (Fabaceae), that is known by has a red resinous exudate from holes in its branches (Daugsch et al., 2008; Piccinelli et al., 2011; Freires et al., 2016; Dantas Silva et al., 2017). The Brazilian red propolis (BRP) can be found in beehives located on the coast of northeastern between states of Bahia, Sergipe, Alagoas, Pernambuco, Paraíba, Rio Grande do Norte, Ceará and Maranhão. The Red propolis from Alagoas state obtained the Geographic Indication (GI) by the Brazilian National Institute of Industrial Property (INPI) (Freires et al., 2016; Dantas Silva et al., 2017). The chemical composition of BRP are rich mainly in isoflavonoids, flavonoids, benzophenones, phenolic acids and terpenes, and has more than 200 compounds as constituents and/or markers (de Mendonça et al., 2015; Freires et al., 2016; do Nascimento et al., 2019). Some pharmacologic activities are attribute to BRP as antibacterial (Sforcin and Bankova, 2011; Bueno-Silva et al., 2013; Dantas Silva et al., 2017; da silva Barboza et al., 2021), anti-inflammatory (Lima Cavendish et al., 2015; Franchin et al., 2016; Corrêa et al., 2017) and wound healing (Jacob et al., 2015; Corrêa et al., 2017; Oryan et al., 2018).

Propolis has been used in dentistry and oral healthcare due to its pharmacological properties and absence of toxicity ensured even by its use in traditional medicine (Sforcin and Bankova, 2011; da silva Barboza et al., 2021; Zulhendri et al., 2021). Among the possibilities of propolis use in dentistry and oral healthcare are: in inhibition of adhesion and biofilm formation of *Candida species* in dentistry materials (Bezerra et al., 2020), on development of endodontic irrigants (Parolia et al., 2021), on development of varnishes (De Luca et al., 2014), on development of total-etching adhesive system (Porto et al., 2021), as cavity cleaning agent (Celerino de Moraes Porto et al., 2018), on mouthwash development (Santiago et al., 2018), on gel formulation development (González-Serrano et al., 2021) and on oromuco-adhesive films development (Arafa et al., 2018).

Composite resins are dental restorative materials that contain an organic matrix, inorganic fillers, bonding agents and a photoinitiator system (Ferracane, 2011). The organic matrix of composite resin content mainly dimethacrylate monomers as bisphenylglycidyl BisGMA, triethylene glycol dimethacrylate (TEGDMA), ethoxylated bisphenol-A dimethacrylate (BisEMA) and Urethane dimethacrylate (UDMA) (Floyd and Dickens, 2006; Goņçalves et al., 2009). The proportion of each dimethacrylate monomers used influences on formation of cross-links in the 3D structure of the polymer and, consequently, in the mechanical properties of composite (Achilias et al., 2010; Vouvoudi et al., 2015). The viscosity of these composites is established by filler content, and this component is a main responsible for the mechanical strength of composites (Liu et al., 2015). The main filler components used in composite resin are titanium dioxide (TiO_2_), ceramic, and zirconia (ZrO_2_) ceramic in form of micro/nanoparticles (Makvandi et al., 2018). Currently, the development of modified composite resins that have antibacterial activity and compatible mechanic and physical-chemical properties with use has been sought (Makvandi et al., 2016; Wang et al., 2017; Liang et al., 2018; Dias et al., 2019).

In this context, the purpose of this study was to modify a commercial composite resin with Brazilian red propolis to obtaining a BRP enriched composite resin that shows antibacterial activity and compatible mechanic and physical-chemical properties with intend use. The null hypothesis tested in this study is that the BRP addiction into a commercial composite resin no provide antibacterial activity, nor compatible mechanic and physical-chemical properties with intend use.

## Materials and Methods

### Collection of Brazilian Red Propolis and Extracts Obtainment

Brazilian red propolis (BRP) raw material was collected in the city of Marechal Deodoro, Alagoas, Brazil in July 2013 (geographic coordinates south latitude: 9° 42.258′, west latitude: 35° 54.391′ and height of 35.5 m above sea level). The access and transportation of Brazilian red propolis was previously authorized by regulatory agencies for control of Brazilian Genetic Heritage and Biodiversity Conservation with protocol number of acceptance 010124/2012-8. The Brazilian red propolis ethanol extract (EBRP) was obtained by extraction of BRP (250 g) through maceration method where raw propolis was manually ground and placed in glass flask with 600 ml of 80% ethanol (v/v) under agitation for 48 h. After this time, the macerate (liquid portion) was removed using a pipette, placed in another glass flask and the resinous mass deposited at the deep of firth glass flask was submitted to new extraction with more 600 ml of 80% ethanol at the same conditions. After the end of 2 cycles of extraction, the total macerate was joined and concentrated in a rotary evaporator (Fisatom, Brazil) in a vacuum at 40°C. The EBRP was then placed in a glass container and left for approximately 3 days for the residual solvent to evaporate. As a result, a solid mass (162 g) with viscous appearance was obtained.

A liquid-liquid extraction of EBRP was carried with hexane and ethyl acetate to eliminate grease and waxes and to concentrate flavonoids, respectively. EBRP (20 g) was solubilized with 60 ml of 70% ethanol (v/v) and successively fractioned with hexane (100 ml, twice) and right after with ethyl acetate (100 ml, twice) giving rise to the Brazilian red propolis hexane extract (HBRP) and the Brazilian red propolis ethyl acetate extract (EABRP), respectively. The EABRP was concentrated in a rotary evaporator to obtain a solid mass (4.0 g) and stored in a freezer at −20°C until further analysis.

### Chemical Characterization and Antibacterial Assay of Brazilian Red Propolis Extracts

#### Analysis of Red Propolis by LC-ESI-Orbitrap-FTMS

The LC-MS analysis was performed using a LC-Orbitrap-FTMS system consisted of an Accela 600 HPLC system combined with an Exactive (Orbitrap) mass spectrometer (Thermo Fisher Scientific, Bremen, Germany) including on-line DAD (200–600 nm) and UV at 280 nm analysis. The chromatographic separation of the compounds from red propolis was performed using an ACE^®^ C18 columns (100 × 4.6 mm; 3 µm) from (Hichrom, Reading United Kingdom) and a mobile phase (0.1% of formic acid in H2O: 0.1% of formic acid in Acetonitrile; A:B) in flow rate of 300 μl/min. The gradient elution was programmed as follows: 0–6 min linear gradient 30–45% of B, 6–14 min linear gradient 45–75% of B, 14–20 min linear gradient 75–100% of B, 20–51 min at 100% of B for elution of the guttiferones and cleaning of the column, 51–54 min decreasing in B to 45%, 54–55 linear gradient 30% of B, 55–60 min isocratic condition with 30% of B to re-equilibration of the column for next run. The injection volume was 10 µl. The MS detection range was from 100 to 1200 m/z set at 30,000 resolutions and the scanning was performed under ESI negative polarity mode with capillary temperature was 250°C.

#### Analysis of Red Propolis by UPLC-PDA

An ultra-performance liquid chromatography coupled with a photodiode array (UPLC-PDA) analysis was performed using an UPLC-PDA system from Shimadzu (Tokyo, Japan). The UPLC-PDA equipment consisted of the modules: degasser (model DGU-20A3R), two high-pressure pumps (model LC-20ADXR), auto-injector (model SIL-20AXR), oven chromatographic column (Prominence^®^ CTO-20A), photodiode array detector (model SPD-M20A), a controller (model CBM-20A), and Shimadzu Labsolution software. The chromatographic separation of flavonoids from red propolis was performed using a Kinetex^®^ C18- column (150 mm × 4.6 mm; 5 µm) and a mobile phase that consisted of solvent A (Milli-Q water) and solvent B (acetonitrile) pumped at flow rate of 0.3 ml/min. The initial elution gradient consisted of 70% water (A) and 30% acetonitrile (B) (v/v). The column was eluted by varying the percentage of (B) as follows: 0–2 min 30% B, 2–5 min 36% B, 5–8 min 46% B, 8–11 min 52% B, 11–14 min 52% B, 14–17 min 57% B, 17–20 min 62% B, 20–24 min 62% B, 24–28 min 68% B, 28–32 min 72% B, 32–36 min 90% B, 36–42 min 97% B, 42–50 min 100% B, 50–55 min 100% B, 55–57 min acetonitrile was reduced to 30% and this condition was maintained up to 60 min. Volumetric working solutions of analytical standards of the daidzein, liquiritigenin, pinobanksin, isoliquiritigenin, formononetin, pinocembrin, and biochanin A from Sigma-Aldrich (St. Louis, MO, United States) was prepared at 200 μg/ml (ethanolic solutions). After that, the working solutions were diluted to obtain a calibration curve at the concentrations: 7.50, 5.00, 2.50, 1.00, 0.50 and 0.15 μg/ml (*n* = 3). This calibration curve was used for the quantification of the listed flavonoids in the EABRP dried extract. The EABRP lyophilized extract (EABRP dried extract) were solubilized in ethanol (HPLC grade) to obtain stock solution at 10 mg/ml. Then, 250 µl of EABRP stock solution was pipetted to 5 ml volumetric flasks (*n* = 3) to obtain work solutions at 500 μg/ml. The injection volume of each sample was 2.0 µl. The UPLC-PDA method was previously validated to the quantification of markers from Brazilian red propolis (do Nascimento et al., 2016). The analysis was carried out at 280 nm.

#### Antibacterial Activity of Brazilian Red Propolis Extracts

The EBRP and EABRP were tested against *Streptococcus mutans* CCT 3440 from Tropical Cultures Collection (CCT). The strain was provided by André Tosello Foundation (Campinas, São Paulo, Brazil). The stock solutions of EBRP and EABRP were prepared at 30 mg/ml in acetone and diluted, first to 10 mg/ml in 1.0% dimethyl sulfoxide (DMSO) and after to 2500 μg/ml (1.0% DMSO). This dilution process was performed to minimize the acetone’s antibacterial action in the assay. The final solutions of EBRP and EABRP (2500 μg/ml in 1.0% DMSO) was made in the same day of the broth microdilution assay. The *S. mutans* CCT 3440 strain was activated in BHA (brain heart infusion agar) for 18–20 h at 37 ± 1°C in a microaerobic environment. The broth microdilution assay was performed to determination the minimal inhibitory concentration (MIC) of BRP extracts. The broth microdilution assay was carried out in microplates (96 wells) and the procedure according to the Clinical and Laboratory Standards Institute (CLSI, 2012) and Rufatto et al. (Celerino de Moraes Porto et al., 2018). The microplates were prepared with 80 µl of brain heart infusion broth (BHI) and 80 µl of EBRP and EABRP at 2500 μg/ml in each well groups from line A of the microplates, respectively. Serial two-fold dilutions was carried out to obtaining ethanol Brazilian red propolis and ethyl acetate Brazilian red propolis extracts at range from 1000 μg/ml to 7.81 μg/ml, respectively (*n* = 6). The inoculum was prepared by direct colonies suspension in sterile saline after strain activation period. A bacterial suspension was standardized equivalent to 0.5 McFarland standard suspension by turbidity comparison, resulting in a bacterial suspension containing approximately 1 × 10^8^ colony-forming units (CFU)/ml. The standardized inoculum suspension containing about 1 × 10^6^ CFU/ml was performed by dilution (1:20) of the 1 × 10^8^ CFU/ml bacterial suspension in sterile saline. Exactly 20 µl of the standardized inoculum suspension were added in each well, resulting a final bacterial concentration of 1 × 10^5^ CFU/ml, according CLSI (CLSI, 2012). The negative controls were A (inoculum + medium) and B (inoculum + medium + 1.0% DMSO). The positive control was inoculum + medium + 0.12% chlorhexidine gluconate. The sterility control test was performed for BHI medium. The microplates were incubated in ambient air at 35 ± 2°C for 20 h. After microplates’ incubation, 5.0 µl of each well was pipetted and plated in Petri dishes containing sterile BHA for minimal bactericidal concentration (MBC) determination after new incubation period of 24 h at 35 ± 2°C. Exactly 20 µl of resazurin (7-hydroxy-10-oxidophenoxazin-10-ium-3-one) solution at 0.15 mg/ml and pH 7.4 (50 mM) were added in each well of the microplates that were incubated for more 2 h at 35 ± 2°C for observation of the bacterial growth. MIC values were defined as the lowest concentration where the well’s color remained purple. The MBC values were defined as the lowest concentration where it did not colony growth visually.

#### Radical (DPPH)^•^ Scavenging Capacity (%RSC–DPPH^•^)

The radical scavenging capacity of the extracts were mensurated by DPPH^**•**^ method following the procedure described by Oliveira et al. (2020), with some modifications. Stock solutions of EBRP and EABRP were performed at 1.0 mg/ml in absolute ethanol. Aliquots of EBRP were transferred to volumetric flask of 5.0 ml, and then 2.0 ml of DPPH solution (39.432 μg/ml; in ethanol) was added obtaining final concentrations of 0.5, 1.0, 1.5, 2.0, 5.0, 10.0, 15.0, 20.0 and 25.0 μg/ml, respectively (*n* = 3). The EABRP samples were obtained in the same way, but to final concentrations of 0.5, 1.0, 1.5, 2.0, 3.0 and 4.0 μg/ml (*n* = 3). The scavenging reaction was performed in dark at 25°C for 30 min. After reaction time, the absorbance readings were performed with a spectrophotometer (Model UV-1240, Shimadzu, Kyoto, Japan) at 518 nm and the solvent ethanol was used as blank. The percentage of radical-scavenging capacity (%RSC—DPPH^•^) of samples was calculated as follows:

$$\text{\%RSC }=\;\left(\left(A_{DPPH}\;–\;A_{sample}\right)\;/\;A_{DPPH}\right)\text{ * }100\sqrt{a^{2}+b^{2}}$$

where *A*_*DPPH*_ is the absorbance of the diluted DPPH solution (2.0 ml of DPPH at 39.432 μg/ml to 5 ml of ethanol); *A*_*sample*_ is the absorbance of the tested sample at specific concentration. The IC_50_ was determined for EBRP and EABRP through of linear equations from its calibration curves y = ax + b, plotted by extract concentration *vs.* %RSC; where: x is the equivalent concentration to IC_50_ of %RSC; y is the numeric value 50; a is the angular coefficient (slope) of specific line; b is the linear coefficient of specific line. The positive control was trolox (6-hydroxy-2,5,7,8-tetramethyl-3,4-dihydrochromene-2-carboxylic acid) (Sigma-Aldrich) at final concentration range from 0.25 to 4.0 μg/ml in ethanol.

#### Total Flavonoids Content

The total flavonoid content (TFC) was determined by AlCl_3_ method (Woisky and Salatino, 1998), with some modifications. Therefore, were performed methanolic stock solutions of 5% AlCl_3_ (w/v), EABRP at 10 mg/ml and quercetin (Sigma-Aldrich) at 1.0 mg/ml, respectively. The assay was performed by add of aliquots of 10 mg/ml EABRP and 100 µl of 5% AlCl_3_ in a 5 ml volumetric flask (methanol). The final concentration range of EABRP was from 50 to 200 μg/ml (*n* = 3). The solutions absorbances were measured in a UV-Vis spectrophotometer set at the 425 nm wavelength after 30 min of incubation in dark room at 25°C. Total flavonoids content was calculated using the quercetin calibration plot (Y = 0.0711x + 0.0027, *R* ^2^ = 0.999) and expressed as mg quercetin equivalent (QE) g^−1^ of dried EABRP extract.

### Preparation of Experimental Composite Samples

The composite resin Filtek™ Bulk Fill Flow (3M/ESPE, St. Paul, MN, United States) color A2 was modified by addition of ethyl acetate Brazilian red propolis (EABRP) in 10 µl of 2-hydroxyethyl methacrylate (HEMA solvent) from Sigma-Aldrich (St. Louis. MO, United States) for development of experimental groups. The composition of the commercial composite is described in Table 1. The commercial composite was used as a control group (RC). To test the HEMA solvent effect alone, 10 μl of pure HEMA was added to commercial composite to obtaining the solvent control group (RS). The two controls were used at all characterization assays of enriched composites with Brazilian red propolis. The final concentrations of EABRP in the experimental composites were 0.10, 0.15, and 0.25% (w/v) to obtaining the experimental groups RP10, RP15 and RP25, respectively.

**TABLE 1:** Filtek™ bulk fill flow (RC) composition.

Lot	Color	Organic matrix % (w/w)	Filler % (w/w)	Photoinitiators % (w/w)
#1827100773	A2	UDMA 10-20	Treated silanized ceramics 50-60	Benzotriazole < 1
		Substituted dimethacrylate 10-20		EDMAB < 1
		Bis-EMA 1-10	YbF_3_ 1-10	
		Bis-GMA 1-10		
		TEGDMA < 1		

UDMA, urethane dimethacrylate; Bis-EMA, bisphenol A ethoxylate dimethacrylate; Bis-GMA, Bisphenol A glycerolate dimethacrylate; TEGDMA, triethylene glycol dimethacrylate; YbF_3_, Ytterbium fluoride; EDMAB, Ethyl 4-(dimethylamino)benzoate.

Three EABRP stock solutions were prepared at concentrations of 10.0, 15.0 and 25.0 mg/ml in HEMA solvent, and the mixture was homogenized by vortex in a dark room. The enriched composites resins groups were obtained by the addition of 10 μl of the EABRP-HEMA solution in 100 mg of the commercial composite resin and photoactivated for 20 s using a curing light (LED Emitter A FIT, 1250 mW/cm^2^, λmax 455 nm, light tip 8 mm, Schuster, Santa Maria, BRA).

The samples of experimental composites groups were prepared in molds with specific dimensions for each type of biological, mechanical, or physicochemical assays and positioned between two polyester strips. Before photoactivation, the upper surface of samples was covered with a glass blade and subjected to a load of 500 g for 1 min to remove excess material and ensure standardization of samples. The glass blade was removed to carry out the photoactivation. The Schematic representation of the process of obtaining and characterization of composite resin enriched with Brazilian red propolis is show in Supplementary Figure S2.

### Biologic Characterization of Composite Resin Enriched With Brazilian Red Propolis

#### Antibacterial Activity (Direct Contact Test)

Five disc-shaped samples to each experimental group were prepared with the aid of a stainless metallic matrix (h = 1.0 mm, Ø = 5.0 mm) and then were photoactivated for 20 s, as describe previously. The antibacterial activity of experimental composites was determined in relation to the *Streptococcus mutans* CCT 3440 strain. The bacterial strain was activated in sterile BHI medium at 37°C for 24 h, and then sowed in BHA medium and incubated at 37°C for 18 h. The inoculum concentration was adjusted in sterile physiologic solution from colonies isolated in BHA. The inoculum was adjusted to 10^6^ UFC/ml for the determination of antibacterial activity by direct contact test.

The antibacterial activity of experimental composite RP25 was also determined by direct contact test (Tavassoli Hojati et al., 2013; Zhang et al., 2017). Therefore, 10 μl of suspended bacteria (10^6^ UFC/ml) were placed on each disc-shaped sample (ø = 5 mm; thickness = 1.0 mm) individually positioned in sterile microtubes (*n* = 5). The disc-shaped sample in contact with inoculum were maintained laminar flow, for 1 h, until the entire liquid suspension evaporated to obtain a thin layer of bacteria. After this period, 200 μl of sterile physiologic solution was added to the each microtubes and the bacterial suspension was homogenized in vortex. To estimate colony formation, bacterial suspension from each specimen was diluted in sterile physiologic solution at 1:10 (10^−1^) proportion, then 25 μl of this dilution was inoculated in BHA plates for 24 h at 37 ± 1°C in a microaerobic environment. After incubation, the total colonies were counted (*n* = 5) and the determination of UFC/ml were performed to each group, using the equation:

UFC/ml = Nº of colonies x 40 x 10

Where: Nº of colonies is the total colonies present the plate after direct contact test; 40 is the correction factor to 1 ml (25 μl × 40 = 1 ml); 10 is the modulus of dilution used (10^−1^) to UFC/ml count. The group RC (commercial composite) was used as negative control. The group RS (commercial composite with the solvent HEMA) was used to evaluate the solvent effect in enriched composite resins formulations. A microtube with inoculum without composites was used as cell control. Antibacterial activity was expressed by antibacterial ratio, where:

r (%)= [(b − c) / b] x 100

Where r (%) is the antibacterial ratio; b is the average UFC/ml recovered for bacterial control; c is the average of recovered UFC/ml for the experimental group.

#### Cytotoxicity

The *in vitro* cytotoxicity assay was performed for determined the cytotoxicity of BRP enriched composite resin against 3T3 fibroblasts. The composite samples were prepared as describe previously, and 24 h later the samples were immersed in pure Dulbecco’s Modified Eagle Medium (DMEM) medium, incubated for 24 h at 37°C, 5% CO_2_ and 95% relative humidity to obtain the extracts (composite resins extracts in DMEM). The extracts were prepared at the ratio of 1.0, 2.0, 3.0, 5.0 and 6.0 cm^2^ of composite/mL of medium to the experimental groups analyzed RC, RS and RP25 (*n* = 5), according to ISO 10993-12 (International Organization for Standarization, 2006). The samples were taken from the culture medium after the specified time and the obtained extracts were filtered in 0.22 μm membranes (Millipore, Molsheim, France) to ensure sterile conditions.

Fibroblasts of the 3T3 strain (embryonic fibroblasts of mice) were sown in DMEM medium supplemented with 10% bovine fetal serum (SBF), L-glutamine (2.0 μM) and penicillin/streptomycin (0.02 μg/ml) and were kept at 37°C, 5% CO_2_ and 95% relative humidity. The cells were transferred to 96-well plates (200 μl/well—7.0 × 10^3^ cells/ml of complete culture medium) and incubated for 24 h in the same conditions as described previously. After this period, the supernatant was removed and each well with cells received 180 μl of fresh complete DMEM and 20.0 μl of the extract, and the cells were incubated for 24 h under the same conditions of temperature, CO_2_ and humidity. The negative control group (cell control) received only fresh complete DMEM. Cell viability was determined by the MTT assay [3-bromide (3,5-dimetiltiazole-2-yl)-2,5-diphenyltrazzole] 24 h after exposure of cells to composite resins extracts in DMEM. The cells were washed with phosphate buffered saline (PBS) and 23 μl of an MTT solution (5.0 mg/ml in PBS) were added to each well as well as 23 μl of an MTT solution (5.0 mg/ml in PBS), then the cells were incubated again for 4 h under the same conditions. The supernatant was discarded and 150 μl dimethyl sulfoxide (DMSO) was added to each well to dissolve the formazan crystals. After 10 min the optical density of the resulting solution was read at 540 nm in a spectrophotometer (Model UV-1240, Shimadzu, Kyoto, Japan). The mean values of optical density obtained from cells exposed to DMEM were used as a negative control reference (100% cell survival). The cytotoxicity of the tested samples was expressed as the percentage of cell viability in relation to the negative control (100%), (*n* = 5). The *in vitro* cytotoxicity was determined by decrease of the cell viability as the equation:

$$\text{Cell viability \% }=\;\left(100\text{ x }OD_{540}\text{ sample}\right)\;/\;OD_{540}\;cell\;control\;$$

where OD_540sample_ is the optical density of cells after sample treatment and OD_540 cell control_ is the optical density of cells control group.

### Mechanical Characterization

#### Three-Point Flexural Strength (ISO 4049)

Rectangular samples were made for each experimental group (*n* = 10) in a stainless-steel matrix (2 mm × 2 mm x 25 mm) according to the specifications of ISO 4049 (International Organization for Standarization, 2000) and then were photoactivated for 20 s, as describe previously. The photoactivation process was carryout according to the ISO 4049 specifications: one moment of photoactivation for each of the three points on top and bottom samples surfaces. After cure, the samples were kept in distilled water at 37°C for 24 h before test. The test was performed in a Microtensile/Semi-universal OM100 test machine (Odeme Dental Research, SC, BRA) with two rods (2 mm in diameter), mounted parallel with 20 mm between its centres, and a third rod (2 mm in diameter) centred between, and parallel to, the other two. The loading ratio and speed applied on samples was of 50 N/min and 0.75 ± 0.25 mm/min, respectively, according to ISO 4049 until the sample fracture. Flexural strength (FS) was expressed in MPa by equation:

$$\text{FS }=\;3Fl\;/\;2bh^{2}$$

where *F* is the maximum load, in newtons, exerted on the sample; *l* is the distance, in millimeters, between the supports; *b* is the width, in millimeters, of the specimen measured immediately prior to testing and *h* is the height, in millimeters, of the specimen measured immediately prior to testing.

#### Knoop Microhardness

Five disc-shaped samples were made for each group, with the aid of a stainless metallic matrix (h = 1.0 mm, Ø = 5.0 mm) and then were photoactivated for 20 s, as describe previously. The samples were stored in distilled water at 37°C for 24 h before test. Five indentations were performed on the irradiated surface of each sample at the center and more four regions with a load of 50 g for 5 s using an HMV-2000 microhardness (Shimadzu, Tokyo, Japan). The average of five indentations was taken as KHN to each of four samples on each experimental group and the average of four KHN in each experimental group was taken as the KHN to the group. The Knoop microhardness (KHN) was determined by equation:

$$KHN\;=\;P\;/\;CL^{2}$$

where *P* is the load applied on the sample (Kgf); *L* is the length of the longest diagonal of the indentation (mm^2^) and *C* is the indentation constant (0.07028).

### Physicochemical Characterization

#### Post-Cure Depth

The post-cure depth was determined according to ISO 4049 procedure specifications (International Organization for Standarization, 2000). Five cylindric-shaped samples per group were produced with the aid of a cylindrical stainless metallic matrix (h = 6.0 mm, Ø = 2.0 mm) and then were photoactivated for 20 s, as describe previously. Then, the samples were immediately removed from the matrix and the uncured material at the bottom surface was scraped with a plastic spatula. The height of the cylindric-shaped samples was measured with a micrometer (with an accuracy of 0.01 mm) after scraping. The post-cure depth to each experimental groups was determined as the mean of each group samples.

#### Degree of Conversion

The degree of conversion of the experimental groups was analyzed by Fourier transform infrared spectroscopy (FTIR; IRAffinity-1 Model, Shimadzu, Kyoto, Japan) in attenuated total reflection (ATR) mode. The DC% was measuring by change of height of the band at 1638 cm^−1^ (corresponding with the aliphatic carbon double bond absorbance). The aromatic carbon double bond absorbance at 1608 cm^−1^ was used as standard control. Each sample spectrum was acquired with 64 scans, resolution of 4.0 cm^−1^ and using the absorbance mode. Disc-shaped samples were made with the aid of a stainless metallic matrix (h = 1.0 mm, Ø = 5.0 mm) and then were photoactivated for 20 s, as describe previously. Were analyzed non-polymerized samples (*n* = 3), immediately polymerized samples (*n* = 3) and samples with 24 h after polymerization (*n* = 3) to each experimental group and theirs respective FTIR spectra were obtained. The degree of conversion was calculated as follows:

$$\left(\text{DC\%}\right)=\;\left[1-\left(\left({\text{R}}_{cured}\right)\;/\;\left({\text{R}}_{uncured}\right)\right)\right]]\text{x }100$$

where: R = (band at 1638 cm^−1^)/(band at 1608 cm^−1^).

#### Water Sorption and Water Solubility

The water sorption (Wsp) and water solubility (Wsl) in water of the experimental resins were determined according to ISO 4049 specifications (International Organization for Standarization, 2000). Five samples (h = 1.0 mm, Ø = 15.0 mm) per experimental group were prepared in a stainless metallic matrix and were photoactivated at nine points on the upper surface, each point for 20 s. After photoactivation, the samples were arranged in a light-proof container for 24 h and transferred to a desiccator containing silica gel dehydrated (Fischer Scientific^®^, Leicester, United Kingdom), this set was kept at 37°C in an oven. After 22 h, all groups were transferred to a second desiccator, where they were kept for 2 h at 37°C. At the end of the cycle, each sample was weighed on an analytical balance with precision of 0.01 mg (Ohaus^®^ DV314C Discovery, Pine Brook, United States) and replaced in the initial desiccator. The cycles were repeated until a constant mass was reached M1 (μg), so that the weight variability was not greater than 0.1 mg in 24 h period. The samples were measured with digital micrometer (accurate to 0.01 mm, DIGIMESS-110-250, Digimess^®^ Precision Instruments Ltda, SP, BR) in order to determine the area (mm^2^) and volume (mm^3^). Then, the samples were immersed in water at 37 ± 1°C (Fanem^®^ Water Bath 1100, SP, BR), for 7 days. After this period, excess water was removed, and each sample again weighed. The wet mass was recorded as M2 (μg). The design cycles were repeated, as previously described, until the stable weight was reached, corresponding to M3 (μg). The mean values of Wsp and Wsl of each sample were calculated in micrograms per cubic millimeter using equations, respectively:

Wsp = (M2 – M3)/V

Wsl = (M1−M3)/V

#### Roughness Analysis

Disc-shaped samples were made with the aid of a stainless metallic matrix (h = 1.0 mm, Ø = 5.0 mm) and then were photoactivated for 20 s, as describe previously. The average surface roughness (Ra) was determined by an atomic force microscopy (AFM) (Model Multiview 4000TM, Nanonics, Jerusalem, Israel) combined with optical microscope (Model BXFM Olympus, Tokyo, Japan). The AFM system was acoustically isolated, and the instrument was stabilized on an active table of movements. The topography of the samples was determined (256 × 256 pixels) in tapping mode with a scan ratio of 0.3–1.0 Hz in an area of 20 × 20 μm. The probe has a diameter <10 nm, 300 µm cantilever, 30° bend angle, 32 kHz resonance frequency and a Cr coating. Twelve regions per experimental group were analyzed and the average roughness (Ra) was determined using the Software WSxM (Nanotec, Madrid, Spain). The roughness average was calculated as the absolute mean of the heights of the irregularities along the profile as the equation:

$$R_{a}=\frac{1}{N}\overset{N}{\underset{i=1}{\sum }}|z_{i}-\bar{z}|$$

Where: N is the number of sample points, z_i_ is the height of each sample point and $\bar{\text{Z}}\;$ is the average height of the sample points.

#### Thermal Analysis (TGA e DSC)

Five disc-shaped samples were prepared in a stainless metallic matrix (h = 1.0 mm, Ø = 5.0 mm) and powdered to obtain uniform powder. Thermogravimetry analysis (TGA) was performed in a TGA-51H model equipment (Shimadzu, Kyoto, Japan), from 4.5 mg ± 6.6% of each sample, packed in alumina crucible. The heating ratio was 10°C min^−1^ in the range of 25–900°C in nitrogen atmosphere with flow of 50 cm^3^ min^−1^. The equipment was calibrated under the same conditions with calcium oxalate monohydrate pattern. Differential scanning calorimetry analysis (DSC) was performed in DSC model 60 plus (Shimadzu, Kyoto, Japan) from 2.5 mg ± 4.0% of each sample packed in hermetically sealed alumina crucible. The heating ratio was 5.0°C min^−1^ in the range of 30–600°C in nitrogen atmosphere and flow of 50 cm^3^ min^−1^. The equipment was calibrated under the same conditions with indium and zinc.

### Statistical Analysis

The statistical analysis was performed using the software GraphPad prism 9.2.0. The Shapiro-Wilk test was performed to determine the normality distribution of results data, at the level of significance of 95%. The parametric data were analyzed by one-way analysis of variance ANOVA followed by Tukey’s comparisons test (*p* < 0.05). The data of cytotoxicity assay of were analyzed by two-way ANOVA followed by Tukey’s comparisons test (*p* < 0.05). The non-parametric data were analyzed by Kruskal-Wallis test followed by Dunn’s comparisons post-test at the level of significance of 95% (*p* = 0.05).

## Results

### Chemical Characterization and Antibacterial Activity of Brazilian Red Propolis Extracts

**FIGURE 1:**
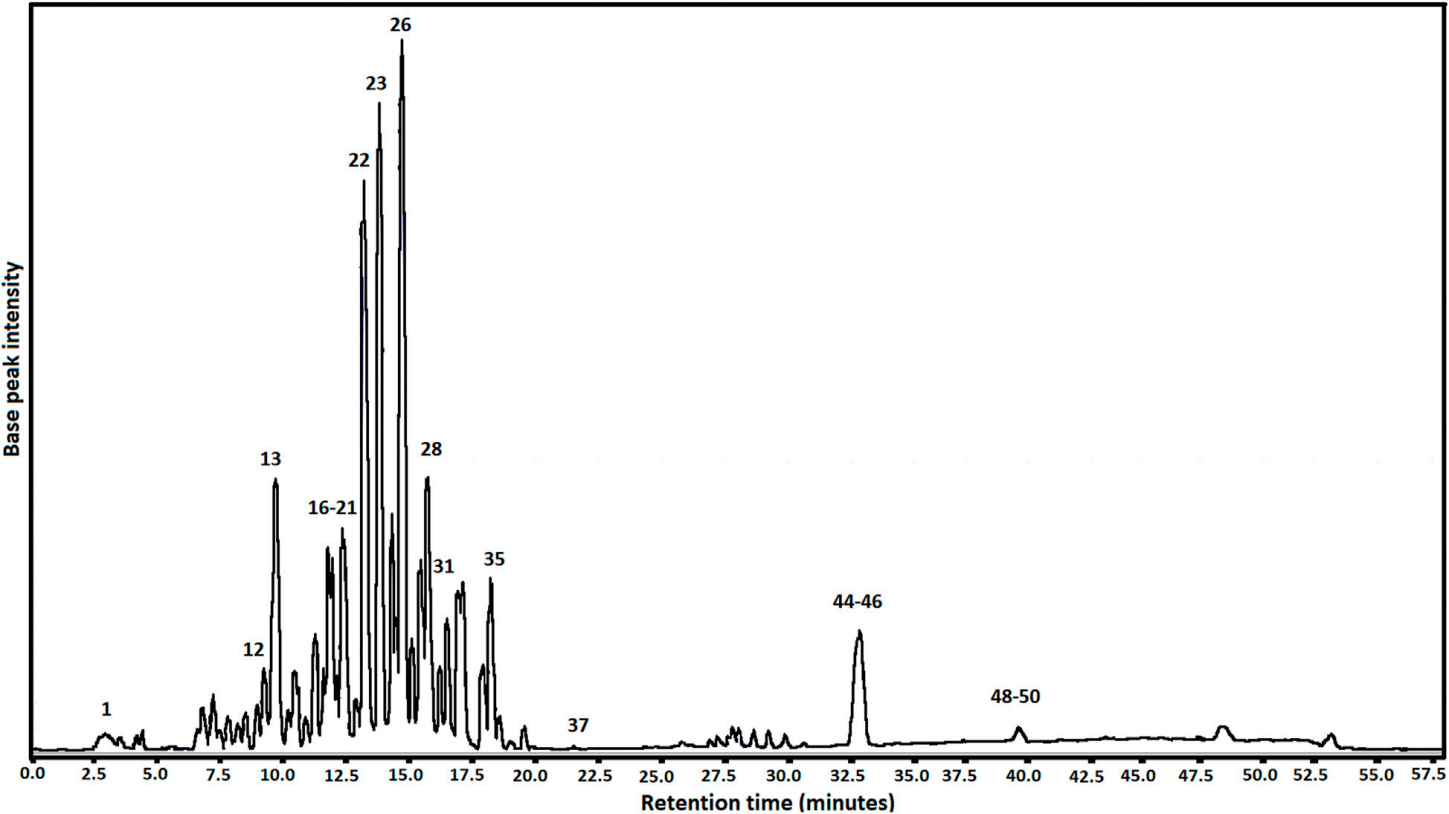
EABRP LC-ESI-Orbitrap-FTMS chromatogram.

The BRP chemical composition was identified by LC-ESI-Orbitrap-FTMS, where was possible to identify fifty compounds from BRP belonging to the class of phenolic acids, flavonoids (isoflavones, isoflavans, flavones, flavanonols, flavonols, flavanols, flavanones, C30 isoflavones, pterocarpan), chalcones, triterpenes and prenylated benzophenones. The EABRP LC-ESI-Orbitrap-FTMS chromatogram shows in Figure 1. Furthermore, is observed that the compounds 4,4′-dihydroxy-2-methoxychalcone (26), formononetin (23), formononetin (22), vestitol (28), liquiritigenin (13), and isoliquiritigenin (21) are the major constituents of this EABRP extract. The identified compounds in the EABRP extract are listed in Table 2. The chemical structures of some of compounds identified from EABRP are show in the Supplementary Figure S1.

**TABLE 2:** Identification and confirmation of some markers of the EABRP using LC-ESI- Orbitrap-FTMS.

Peak	RT (min.)	[M-H]^-^ (m/z)	MW	Formulae	Compound
1	2.95	179.0556	180.16	C_9_H_8_O_4_	Caffeic acid
2	2.98	193.0502	194.18	C_10_H_10_O_4_	Ferulic acid
3	3.00	178.0556	179.05	C_9_H_8_O_4_	Umbelic acid
4	3.04	163.0243	164.16	C_9_H_8_O_3_	p-coumaric acid
5	3.10	475.1232	476.43	C_23_H_24_O_11_	7-O-beta-glucopyranosyl-4′-hydroxy-5-methoxyisoflavone
6	4.50	461.1023	462.40	C_22_H_22_O_11_	6-Methoxyluteolin 7-rhamnoside
7	7.05	269.0811	270.24	C_15_H_10_O_5_	Genistein
8	7.35	285.0395	286.24	C_15_H_10_O_5_	Kaempferol
9	8.04	289.0711	290.27	C_15_H_14_O_6_	Cathechin
10	8.28	287.0553	288.25	C_15_H_12_O_6_	Dalbergioidin
11	8.83	289.0711	290.27	C_15_H_14_O_6_	Epicatechin
12	8.95	253.0499	254.24	C_15_H_10_O_4_	Daidzein
13	9.70	255.0654	256.27	C_15_H_12_O_4_	Liquiritigenin
14	10.5	283.0384	284.26	C_16_H_12_O_5_	2′-Hydroxyformononetin
15	11.3	331.0810	332.30	C_17_H_16_O_7_	Evernic acid
16–17	11.9	271.0602	272.25	C_15_H_12_O_5_	Narigenin/Pinobanksin
18	12.4	285.0758	286.24	C_15_H_10_O_6_	Calycosin
19	12.8	521.1600	522.173	C_32_H_26_O_7_	Retusapurpurin B
20	13.2	521.1600	522.173	C_32_H_26_O_7_	Retusapurpurin A
21	13.4	255.0654	256.27	C_15_H_12_O_4_	Isoliquiritigenin
22–23	13.77	267.0655	268.28	C_16_H_12_O_4_	Formononetin/Isoformononetin
24	15.5	253.087	254.28	C_16_H_14_O_3_	6-Methoxyflavanone
25	15.5	287.056	288.25	C_15_H_12_O_6_	6-Hidroxynaringenin
26	14.2	269.0812	270.28	C_16_H_14_O_4_	4,4′-dihydroxy-2-methoxychalcone
27	14.2	269.0812	270.32	C_16_H_14_O_4_	(7S)-dalbergiphenol
28	14.66	271.0603	272.29	C_16_H_16_O_4_	Vestitol
29	15.10	269.0813	270.28	C_16_H_14_O_4_	Pinostrobin
30	15.10	269.0813	270.27	C_16_H_14_O_4_	Medicarpin
31	16.2	271.0607	272.29	C_16_H_16_O_4_	2′,6′-dihydroxy-4′-methoxydihydrochalcone
32	16.2	283.0657	284.26	C_16_H_12_O_5_	Thevetiaflavone
33	16.42	283.0603	284.26	C_16_H_12_O_5_	Biochanin A
34	16.73	253.0865	254.25	C_15_H_10_O_4_	Chrysin
35	16.87	255.1019	256.27	C_15_H_12_O_4_	Pinocembrin
36	17.0	539.1699	540.56	C_32_H_28_O_8_	3′,4′-di-O-benzyl-7-O-(2-hydroxyethyl)-3-O-methylquercetin
37	17.9	285.113	286.32	C_17_H_18_O_4_	Sativan
38	18.2	285.1131	286.32	C_17_H_18_O_4_	(3S)-7-O-methylvestitol
39	18.2	285.1131	286.32	C_17_H_18_O_4_	7,3′-Dihydroxy-4′-methoxy-8-methylflavane
40	21.4	425.1603	426.71	C_30_H_50_O	Cycloartenol/α-amyrin/β-amyrin
41	23.6	533.2906	534.69	C_33_H_42_O_6_	Hyperibone H
42	25.5	617.3480	618.82	C_38_H_50_O_7_	16-hidroxiguttiferone K
43	27.3	511.1383	512.50	C_30_H_24_O_8_	Rhuschalcone V
44	32.80	601.3533	602.80	C_38_H_50_O_6_	Guttiferone F
45	32.88	601.3533	602.80	C_38_H_50_O_6_	Xantochymol
46	32.90	601.3533	602.80	C_38_H_50_O_6_	Guttiferone E
47	34.10	347.2233	348.52	C_22_H_36_O_3_	Anacardic acid (6-pentadecylsalycilic acid)
48	39.24	669.4355	670.917	C_43_H_58_O_6_	Guttiferone C
49	39.24	669.4355	670.917	C_43_H_58_O_6_	Guttiferone D
50	39.24	669.4355	670.917	C_43_H_58_O_6_	Guttiferone B

RT, Retention time (min); MW, Molecular weight.

**FIGURE 2:**
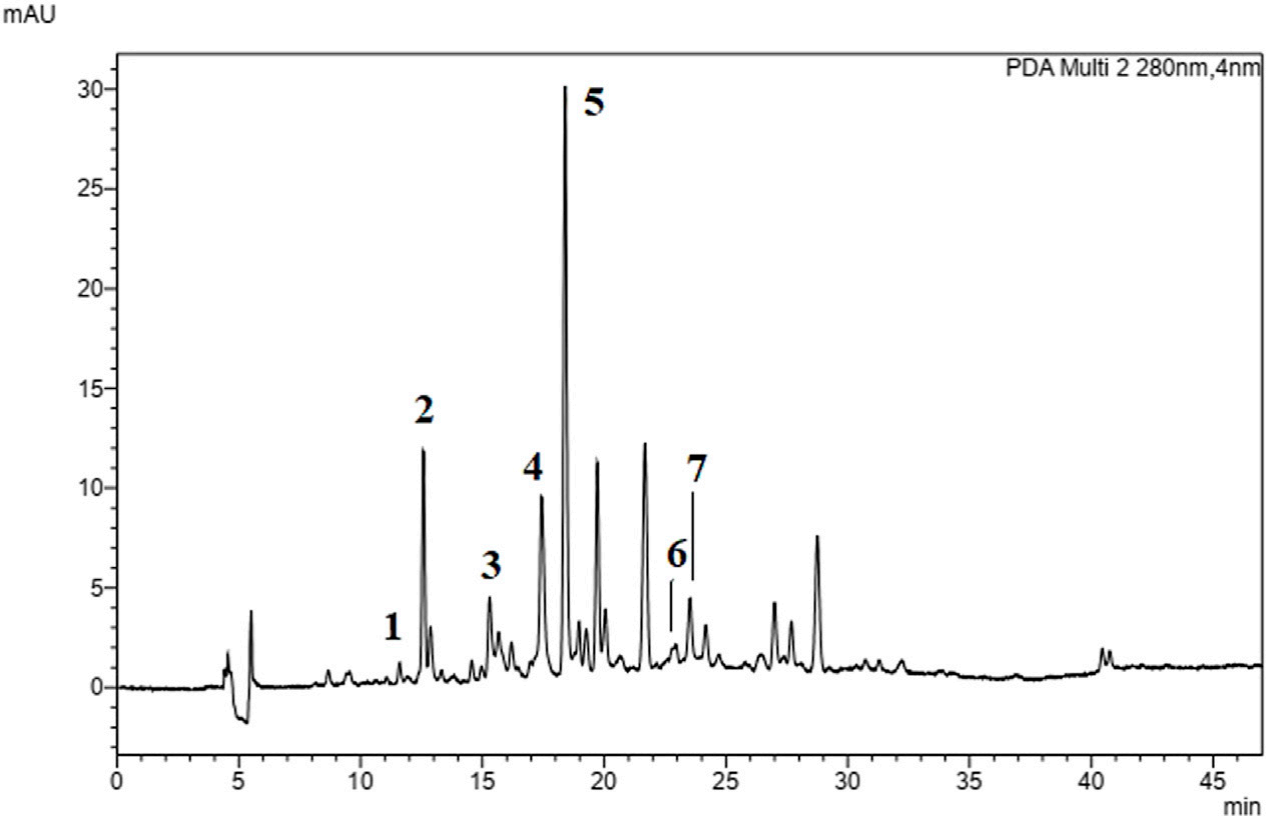
EABRP UPLC-DAD chromatogram at 280 nm. 1 (daidzein), 2 (liquiritigenin), 3 (pinobanksin), 4 (isoliquiritigenin), 5 (formononetin), 6 (pinocembrin) and 7 (biochanin A).

The quantification of seven markers from EABRP was performed by UPLC-DAD. The flavonoids daidzein, liquiritigenin, pinobanksin, formononetin, pinocembrin, biochanin A and the chalcone isoliquiritigenin were identified and quantified from EABRP dried extract. Its respective concentrations are showed in Table 3. The EABRP UPLC-DAD chromatogram too shows formononetin, liquiritigenin and isoliquiritigenin as majority compounds of EABRP dried extract Figure 2.

**TABLE 3:** Quantification of some chemical compound in EABRP by UPLC-DAD.

Peak	Compound	RT (min)	Concentration (µg/ml)	± SD
1	Daidzein	11.60	0.561	0.070
2	Liquiritigenin	12.62	7.797	0.628
3	Pinobanksin	15.66	0.687	0.219
4	Isoliquiritigenin	17.45	4.537	0.501
5	Formononetin	18.40	12.154	0.727
6	Pinocembrin	22.54	0.260	0.040
7	Biochanin A	23.52	0.890	0.198

RT, Retention time (min).

In the present work, the total flavonoids content (TFC) was determined by a colorimetric method using AlCl_3_ reagent. The TFC were expressed as mg quercetin equivalent (mg QE g^−1^) of dried EABRP extract. The EABRP extract contain 54.56 ± 0.25 mg QE g^−1^ of dried EABRP extract Table 4. The radical scavenging capacity (RSC) of EBRP and EABRP were determined by DPPH^•^ method, the EABRP showed RSC fifteen times bigger than EBRP (*p* < 0.05) Table 4. The more capacity radical scavenge verified to EABRP was expected because the liquid-liquid extraction leads to it obtain as a more flavonoids enriched extract. The RSC to EABRP not showed statistical difference in comparation with the RSC to the trolox standard (*p* > 0.05). One of characteristics of a flavonoid enriched extract is precisely its radical scavenging capacity.

**TABLE 4:** Radical scavenge capacity, total flavonoids content, minimal inhibitory concentration and minimal bactericidal concentration of BRP extracts.

Sample	RSC IC_50_ (μg ml^−1^)	TFC (mg QE* g^−1^ dry extract)	MIC (μg ml^−1^)	MBC (μg ml^−1^)
	(Mean ± SD)	(Mean ± SD)	S. mutans** (Mean ± SD)	*S. mutans* ^**^ (Mean ± SD)
EABRP	1.01 ± 0.73^a^	54.56 ± 0.25	125 ± 0.00	500 ± 0.00
EBRP	15.63 ± 1.22^b^	-	125 ± 0.00	1000 ± 0.00
Trolox	2.13 ± 1.33^a^	-	-	-

*Quercetin equivalent.

**Antibacterial activity of BRP extracts against *Streptococcus mutans* CCT 3440.

-, not determined.

Different lowercase letters indicate the statistical difference (*p* < 0.05) between the experimental groups.

EBRP, Ethanolic Brazilian red propolis; EABRP, Ethyl acetate Brazilian red propolis; RSC, Radical scavenge capacity against DPPH ^•^; TFC, Total flavonoids content; MIC, minimal inhibitory concentration; MBC, minimal bactericidal concentration.

The antibacterial activity of EBRP and EARP against *Streptococcus mutans* CCT 3440 was determined by broth microdilution assay where the minimal inhibitory concentration (MIC) and the minimal bactericidal concentration (MBC), were determined to each extract. The MIC values to EBRP and EABRP were of 125 μg/ml, being both characterized as bacteriostatic extracts at this concentration for this bacterial strain. The EABRP extract showed MBC value of 500 μg/ml, being twice more active than EBRP extract that showed MBC value of 1000 μg/ml, Table 4.

### Biologic Characterization of Composite Resin Enriched With Brazilian Red Propolis

Brazilian red propolis enriched composite resins were developed by the addiction of EABRP extract at the concentrations of 0.10%, 0.15%, and 0.25% (w/w) in a commercial composite resin to obtaining the formulations RP10, RP15 and RP25, respectively. The biologic characterization of enriched composites was carried out for determination of the antibacterial activity against *Streptococcus mutans* CCT 3440 and the cytotoxicity to 3T3 fibroblasts, respectively.

**FIGURE 3:**
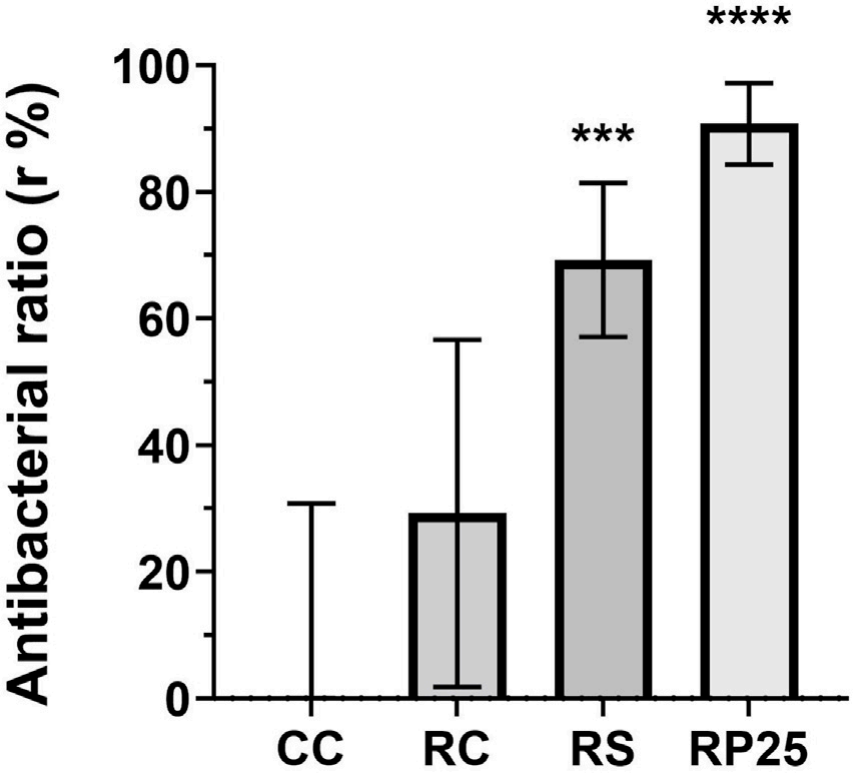
Antibacterial ratio (r %) of EABRP enriched composite resin against *S. mutans* after 1h of direct contact test. CC, cell control; RC, commercial resin; RS, commercial resin + HEMA solvent. RP25, enriched EABRP composite resin at 0.25% (w/w). ***: *p* = *0*.002 ****: *p* < 0.0001.

The antibacterial activity of Brazilian red propolis enriched composite resins was performed by direct contact test and the antibacterial ratio (r %) was determined Figure 3. The statistical results shows that the antibacterial ratio of RP25 was 90.76 ± 6.43 (*p* < 0.0001) and that negative control (RC) did not exhibit significant antibacterial ratio (*p* = 0.1865) after 1 h of direct contact, both in comparison with cell control. The addition of EABRP to the commercial composite resulted in an enriched composite and with antibacterial activity against *S. mutans* CCT 3440 after 1 h of direct contact. The use of methacrylate’s monomer hema as solvent for incorporate propolis to commercial composite in the development phase contributed to the antibacterial activity verified to RP25. This fact is confirmed by antibacterial activity exhibit for RS with an antibacterial ratio of 69.23 ± 12.16 (*p* = 0.0006) when compared with cell control. When comparing RP25 and RS with RC, separately, both shows statistical difference in relation of this commercial composite resin (*p* = 0.0019 and *p* = 0.0454, respectively) Table 5. Thereby, the null hypothesis was discarded and considered the test hypothesis that the addition of EABRP in hema in commercial composite resin led to obtainment of a EABRP enriched composite resin with antibacterial activity.

**TABLE 5:** Antibacterial ratio and cytotoxicity of Brazilian red propolis enriched dental composite.

Sample	r (%)	Cell viability (%) at dose of 3.0 cm^2^ of dental composite/mL
	(Mean ± SD)	(Mean ± SD)
CC	0.00 ± 30.76	100 ± 0.20
RC	29.23 ± 27.41^ns^	79.82 ± 13.12^c^
RS	63.23 ± 12.16***	86.8 ± 13.38^c^
RP25	90.76 ± 6.43****	117.05 ± 18.84^a^ ^,^ ^b^

a Statistically significant difference in comparison with RC group, *p* = 0.0143.

b Statistically significant difference in comparison with RS group, *p* = 0.0293.

c no statistically significant difference in comparison between RC and RS, *p* = 0.8981.

^ns^: no statistically significant difference in comparison with cell control.

***: statistically significant difference in comparison with cell control, *p* = 0.0006.

****: statistically significant difference in comparison with cell control, *p* < 0.0001.

CC, cell control (Streptococcus mutans CCT 3440); RC, commercial dental composite; RS, commercial dental composite + hema solvent; RP25, Ethyl acetate Brazilian red propolis extract enriched dental composite at 0.25% (w/w); r (%), antibacterial ratio.

#### Cytotoxicity

**FIGURE 4:**
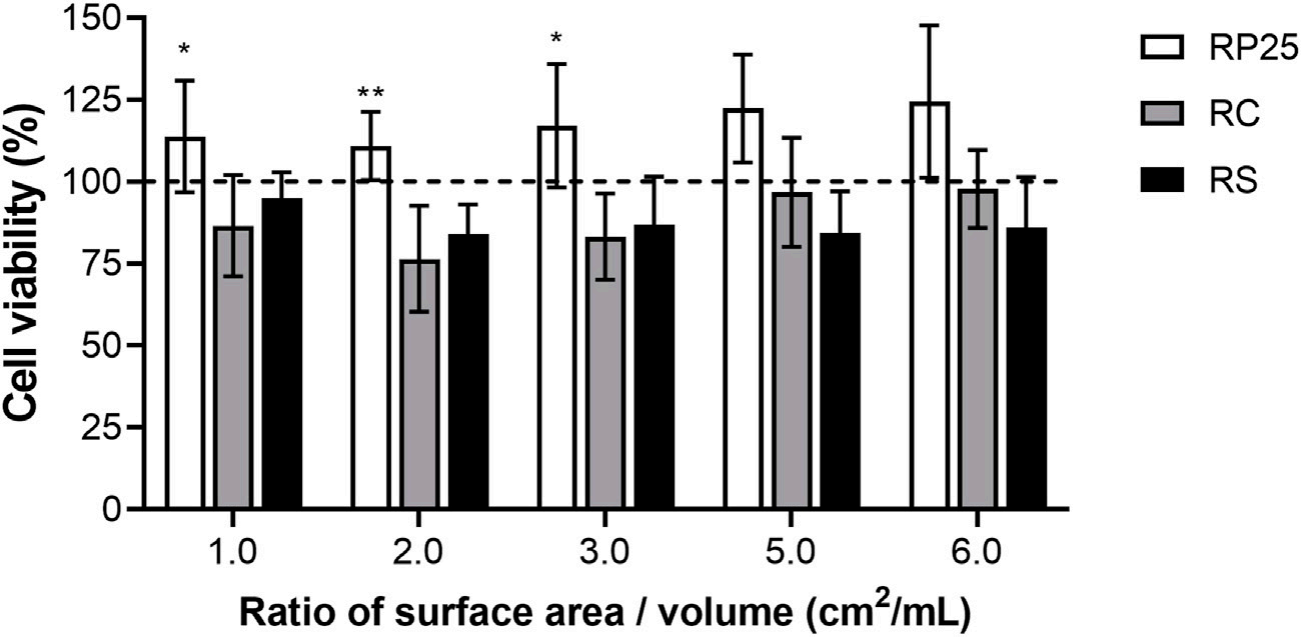
Cytotoxicity of EABRP enriched composite resin against 3T3 fibroblast. Dot line: cell control (100% viability); RC, commercial resin; RS, commercial resin + HEMA solvent; RP25, enriched EABRP composite resin at 0.25% (w/w). *: *p* < 0.05 (comparison between RP and RC).

The cytotoxicity of BRP enriched composite resins was determined by MTT assay against fibroblasts 3T3. In this assay were observed the effect of addition of BRP in the commercial composite resins and the effect of composite resins dose on the cytotoxicity. The usual dose of composite resin for this assay was defined as ratio of 3.0 cm^2^ of composite resin/mL (surface area/volume), conforming ISO 10993-12 (International Organization for Standarization, 2006). The result shows that the groups RP25, RC and RS no presented cytotoxicity to 3T3 fibroblasts, once that after 24 h of exposition to composite resins extracts the cell viability were bigger than 70% Figure 4. Nevertheless, was observed statistically significant difference on the cell viability between experimental groups when analyzed the addition of BRP to the composite resin (*p* < 0.0001). The dose effect was not statistically significant (*p* = 0.0611). Comparing the results for groups RP25, RC and RS at ratio of 3.0 cm^2^ is possible observed that the cells show bigger viability after exposition to RP25 than after to RC and to RS exposition (*p* < 0.05), respectively, as shows Table 5.

### Mechanical Characterization

#### Three-Point Flexural Strength and Knoop Microhardness

**FIGURE 5:**
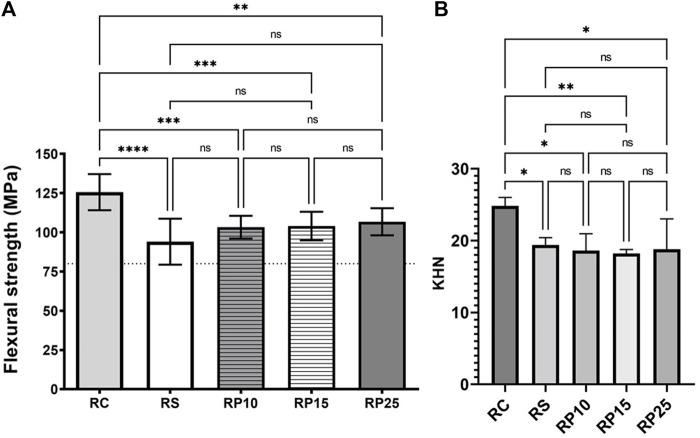
Mechanical characterization of EABRP enriched composite resin. **(A)**: Flexural strength determination (MPa). Dot line: minimal limit of 80 MPa (ISO 4049). **(B)**: Knoop microhardness. RC, commercial resin; RS, commercial resin + HEMA solvent; RP10, RP15 and RP25, enriched EABRP composite resins at 0.10, 0.15 and 0.25% (w/w).

BRP enriched composite resins exhibit flexural strength above of 100 MPa, according to the ISO 4049 specifications (minimal FS of 80 MPa). Formulations RP10, RP15, RP25 and RS are statistically similar (*p* > 0.05) and RC shows the biggest FS 125.57 MPa ± 11.49 (*p* < 0.05) Figure 5A. KHN for RS, RP10, RP15 and RP25 were 19.4 ± 1.5, 18.6 ± 1.3, 18.2 ± 1.3 and 18.8 ± 1.1, respectively Figure 5B. The KHN for commercial composite resin (RC) was 24.8 ± 1.0. The addiction of HEMA, a methacrylate monomer solvent used as diluent in composite resin development was responsible by decrease of the mechanical properties of BRP enriched composite resins compared with RC (*p* < 0.05) once that promotes the decrease of viscosity in comparation. Decrease of viscosity in composite resins is caused by decreased of filler proportion in the composite.

### Physicochemical Characterization

#### Post-Cure Depth

**FIGURE 6:**
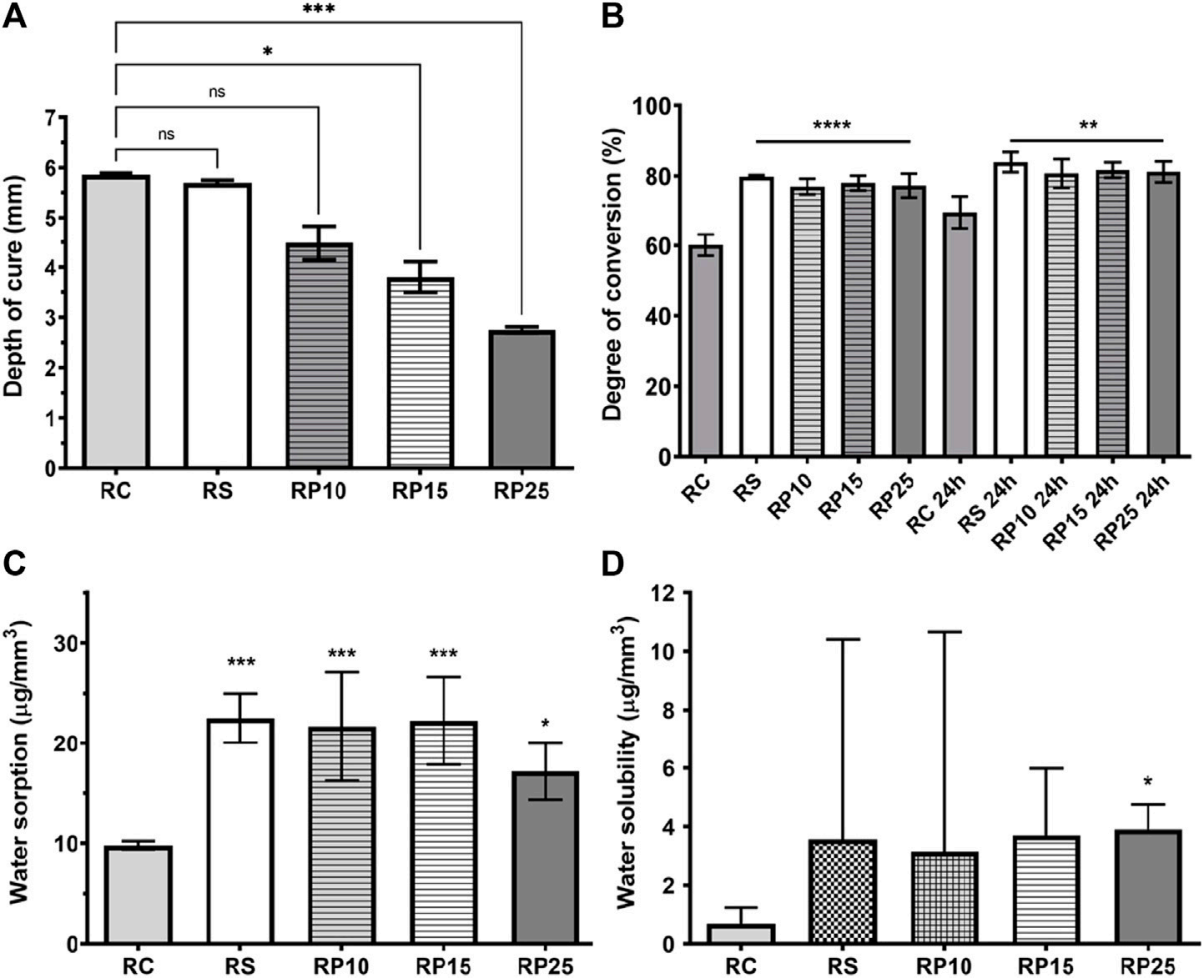
Physical-chemical characterization of EABRP enriched composite resins. **(A)**: Depth of cure. **(B)**: Degree of conversion. **(C)**: Water sorption. **(D)**: Water solubility. RC, commercial resin; RS, commercial resin + HEMA solvent; RP10, RP15 and RP25, enriched EABRP composite resins at 0.10, 0.15 and 0.25% (w/w).

Composites enriched with red propolis showed a cure depth compatible with the commercial composites used as a basis in this study, the bulk composites (with increment of ≥ 4.0 ± 0.1 mm), except RP25. The post-cure depth of RP25 and RP15 was 2.74 ± 0.06 mm and 3.80 ± 0.30 mm, respectively, both smaller than RC (5.86 ± 0.03 mm), RS (5.69 ± 0.05 mm) and RP10 (4.48 ± 0.34 mm) composites Figure 6A. The post-cure depth among the enriched composites was inversely proportional to the red propolis concentration: RP10 > RP15 > RP25. This phenomenon can be explained because the increase of red propolis concentration at composites led to decrease of its translucency, decreasing the capacity of irradiation of the curing light through sample. The Shapiro-Wilk normality test exhibit a non-normal distribution of post-cure depth results; therefore, the Kruskal-Wallis nonparametric test was performed. There was not statistically significant difference between three formulations of enriched composite resins (*p* > 0.05), but there was statistically significant difference between: RP25 and RC (*p* = 0.0002); RC and RP15 (*p* = 0.0171). The statistical analysis demonstrated that there was not significant difference between RC and RP10 (*p* = 0.2546). In this way, RP10 can be considered a bulk enriched composite resin.

#### Degree of Conversion

This study analyzed the degree of conversion of BRP enriched composite resins by FTIR spectroscopy in twice moments: immediately after light curing photoactivation and 24 h after photoactivation. Was observed that the addition of EARP-HEMA to the commercial composite promoted a significant increase in the DC% of enriched composites when compared to the RC Figure 6B. When analyzing each moment separately, is possible verify that at first moment RS, RP10, RP15 and RP25 are statistically similar each other (*p* > 0.05) showing DC% among 76.95 ± 2.24% to 79.81 ± 0.39% and have bigger DC% than RC with 60.17 ± 3.09% (*p* < 0.0001). At the second moment this result profile remains: RS, RP10, RP15 and RP25 are statistically similar each other (*p* > 0.05) showing DC% from 80.70 ± 4.07% to 83.96 ± 2.87% and have bigger DC% than RC with 69.58 ± 4.51% (*p <* 0.029).

#### Water Sorption and Water Solubility

The BRP enriched composite resins, RC and RS controls exhibited values conforming ISO 4049 specification for Wsp (≤40.0 μg/mm^3^) Figure 6C. RS, RP10, RP15 and RP25 were statistically like each other (*p* > 0.05), but smaller than of RC (*p* < 0.028). The Wsl for BRP enriched composite resins was conforming ISO 4049 (≤7.5 μg/mm^3^) Figure 6D. The Shapiro-Wilk test demonstrated that Wsl data have non-normal distribution, therefore was conducted the Kruskal-Wallis test (*p* < 0.05) for this assay. Was observed that RP25 showed a median of 3.9 μg/mm^3^, exhibiting a bigger Wsl than RC with 0.68 μg/mm^3^ (*p* = 0.0109). The groups RC, RS, RP10 and RP15 no showed statistically significant difference (*p* > 0.05) each other.

#### Average Roughness

**FIGURE 7:**
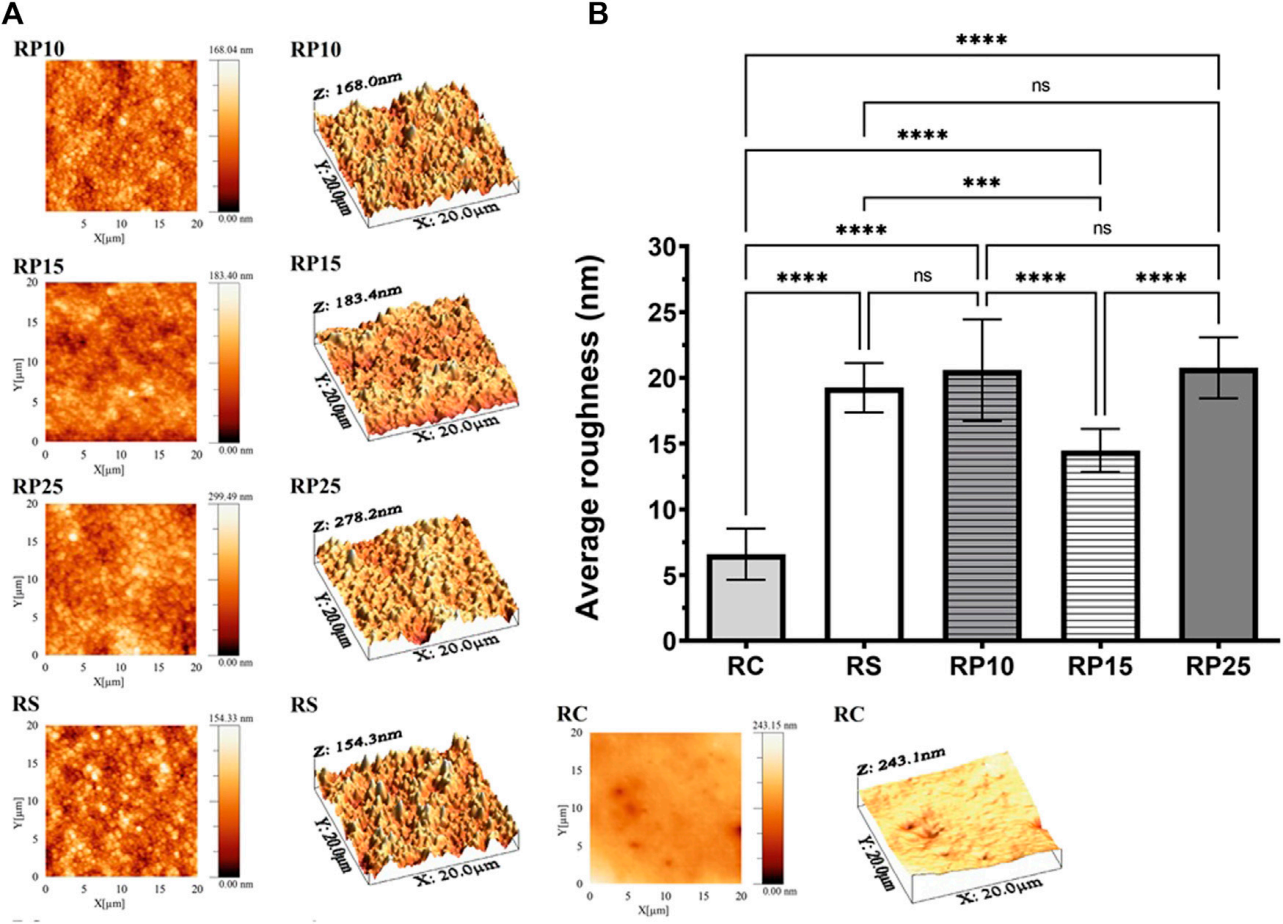
Average roughness (Ra) of EABRP enriched composite resins. **(A)**: Topographic images obtained by AFM for experimental groups. **(B)**: Average roughness values (Ra) for all resins analyzed. RC, commercial resin; RS, commercial resin + HEMA solvent; RP10, RP15 and RP25, enriched EABRP composite resins at 0.10, 0.15 and 0.25% (w/w).

Topographic images obtained in AFM, representative of the average surface roughness (Ra) of the experimental groups are presented in Figure 7A. The values of Ra for BRP enriched composite resins are presented in Figure 7B and show that all groups presented average surface roughness below 21 nm. RS, RP10, RP15 and RP25 exhibited small Ra than RC (*p* < 0.0001), being RC presented the lowest Ra (6.59 ± 1.94 nm), followed for RP15 (14.48 ± 1.63 nm). There was no significant difference (*p* > 0.05) between the Ra values of groups RS (19.24 ± 1.87 nm), RP10 (20.58 ± 3.86 nm) and RP25 (20.76 ± 2.31 nm).

#### Thermal Analysis (TGA e DSC)

**FIGURE 8:**
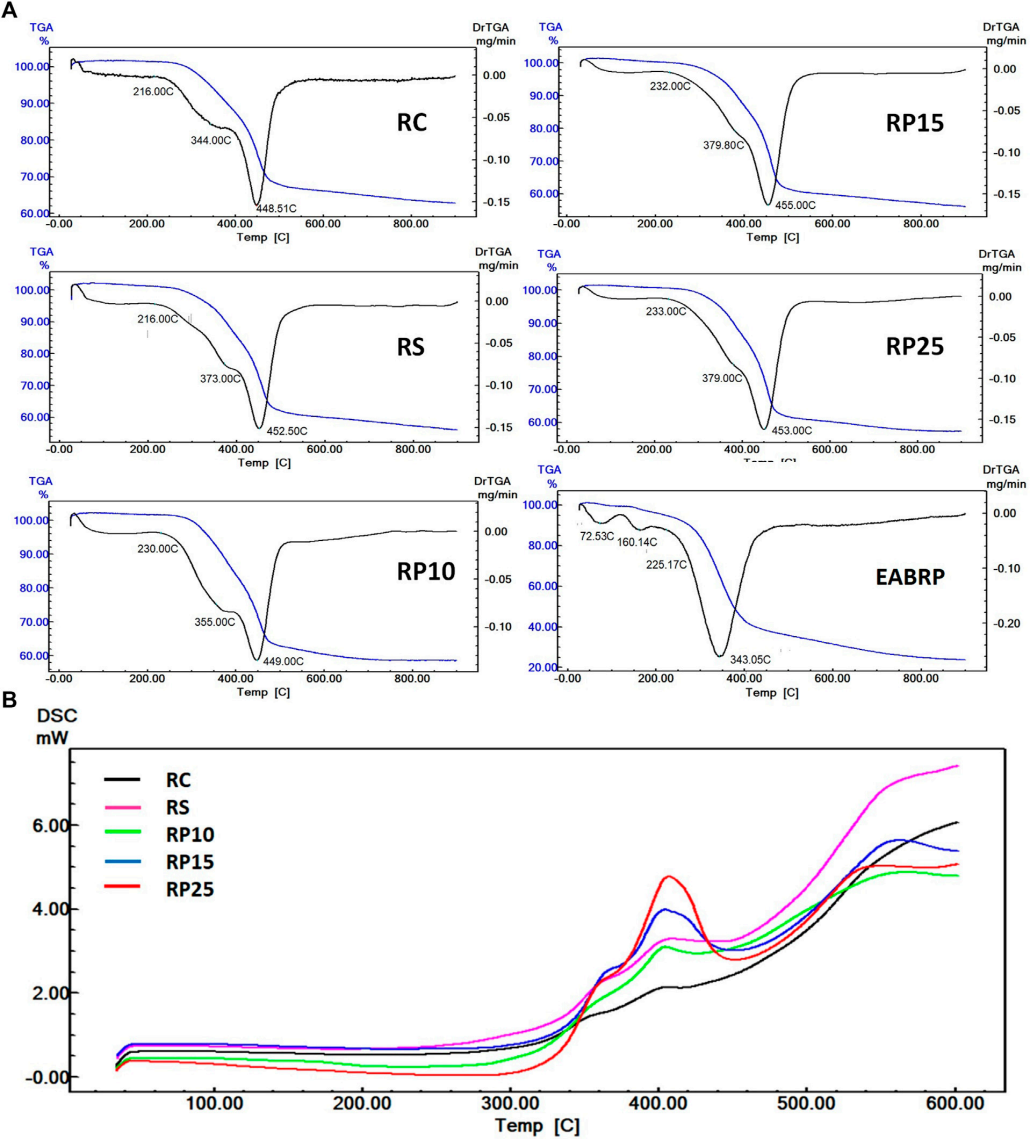
Overlapping thermograms of experimental and control resins. **(A)**: Overlapping TGA and DTG thermograms. **(B)**: Overlapping DSC thermograms.

Was performed the thermal analysis for BRP enriched composite resins for determination of its thermal events of degradation that explain the degree of formation of covalent bonds in the composite polymeric chains. The more resistant to thermal degradation below 600°C, the greater the number of covalent bonds existing in the three-dimensional structure of the composite. The TGA, DTG and DSC thermograms are shown in Figure 8. The thermogravimetric profiles of the studied groups were similar, where all groups presented two events of mass loss, one between 334–379°C and the other between 448–455°C. All composite resins formulations analyzed presented thermal resistance between 300–350°C, since no group had mass loss of up to 5% below these temperatures Figure 8A. The values of thermogravimetric events can be observed in Table 6, where it is observed that the addition of EARP-HEMA led to increased thermal resistance of composite resins.

**TABLE 6:** Thermal resistance, T_0_, T_1_, T_2_ and residual mass at 600°C of EABRP enriched composite resins.

Group	Thermal resistance (ºC)	T_0_ (ºC)	T_1_ (ºC)	T_2_ (ºC)	Residual mass at 600°C(%)
RC	325	216	334	448.5	65.05
RS	340	216	373	452	58.02
RP10	339	230	355	449	58.35
RP15	349	232	379.8	455	58.18
RP25	349	233	379	453	58.73

Thermal resistance, temperature where there is a loss of 5% of the initial mass of the dental composite; T_0_, temperature of onset of thermal degradation; T_1_, primary thermal degradation temperature; T_2_, secondary thermal degradation temperature; RC, commercial resin; RS, commercial resin + HEMA solvent; RP10, RP15 and RP25, enriched EABRP composite resins at 0.10, 0.15 and 0.25% (w/w).

All samples presented similar DSC profile between each other, with two events of thermal decomposition of the polymer matrix in 368°C and 407°C, and the beginning of thermal degradation of the composite stars at ∼ 560°C Figure 8B. It was observed that the addition of EARP-HEMA led to a increase in the heat variation involved in the thermal decomposition of the polymer matrix of enriched composite resins. The values of DSC events can be observed in Table 7.

**TABLE 7:** Thermal decomposition events of EABRP enriched composite resins.

Group	T_1_(ºC)/heat (J/g)	T_2_(ºC)/heat (J/g)	T_3_(ºC)/heat (J/g)
RC	368.24/8.82	422.4/25.01	563.66/9.51
RS	378.49/13.15	407.43/84.48	583.83/152.61
RP10	368.86/9.40	404.65/71.53	565.90/216.52
RP15	375.14/18.70	402.17/216.87	561.69/276.95
RP25	371.85/30.98	405.80/369.55	550.52/206.26

T_1_, First thermal decomposition event; T_2_, second thermal decomposition event; T_3_, Start of thermal degradation of the fillers; RC, commercial resin; RS, commercial resin + HEMA solvent; RP10, RP15 and RP25, enriched EABRP composite resins at 0.10, 0.15 and 0.25% (w/w).

## Discussion

The Ethyl acetate Brazilian red propolis extract (EABRP) was chose for enriched composite resin development because its antibacterial activity against *Streptococcus mutans* CCT 3440. Main idea in development of BRP enriched composite resin was the obtain of a antibacterial composite resin against *Streptococcus mutans*, the primary etiologic agent of caries (Koo et al., 2003). Phenolic acids, flavonoids, chalcones, triterpenes and prenylated benzophenones detected by LC-ESI-Orbitrap-FTMS in BRP extracts are characteristic for Brazilian red propolis collected in the northeast Brazil region (Frozzada et al., 2013; López et al., 2014; de Mendonça et al., 2015; Freires et al., 2016; Rufatto et al., 2018). The flavonoids formononetin, biochanin A, daidzein, liquiritigenin and the chalcone isoliquiritigenin, quantify on this study, are some of markers describes for BRP (Andrade et al., 2017; Picolotto et al., 2019). The total flavonoid content for EABRP observed in this work is bigger than ethanol BRP extract describes in another work (31.48 mg QE g^−1^) because the liquid-liquid extraction carried out concentrated the flavonoids in the acetate phase (Andrade et al., 2017).

The antibacterial results observed for EABRP against *S. mutans* was compatible with results in another studies where ethanol extract of BRP was tested against *S. mutans* UA159 and exhibited MIC and MBC values of 200.0 μg/ml for both assay (Bueno-Silva et al., 2013). In another study chloroform extract of BRP exhibited MIC and MBC against *S mutans* of 125 and 500 μg/ml, respectively (Oldoni et al., 2011). In a study, the antibacterial activity of eight ethanolic Brazilian propolis extracts from 3 types (red, green, and brown) was analyzed against *Enterococcus* sp. ATCC 2912, *Staphylococcus aureus* ATCC 25923 and *Klebsiella* sp. ATCC 1706/700603, and red propolis showed the highest antibacterial activity against this bacteria with MIC values of 31.3, 62.5, and 31.3 μg/ml, respectively (Dantas Silva et al., 2017). These variations in antibacterial results for Brazilian red propolis from the same origin can be explained by seasonality. It is known that the antibacterial activity of propolis may vary depending on seasonality because the presence and concentration of its markers are related with the season, which in turn influences the production of secondary metabolites by the botanical origin of propolis (Regueiro et al., 2017; do Nascimento et al., 2019).

The use of propolis on technological development in dentistry and oral health has been frequent because it is a nontoxic raw material, with traditional use in natural medicine and that shows some known pharmacological activities as antimicrobial, anti-inflammatory and wound healing (Koo et al., 2002; Freires et al., 2016; Oliveirados et al., 2017; de Carvalho Furtado et al., 2018; Bezerra et al., 2020; da silva Barboza et al., 2021). It has been reported in scientific literature the use of propolis on development of varnish (De Luca et al., 2014; Neto et al., 2020), toothpaste (Wiatrak et al., 2017; Peycheva et al., 2019), total-etching adhesive system (Porto et al., 2021), cavity cleaning agent (Celerino de Moraes Porto et al., 2018) and endodontic irrigant (Parolia et al., 2021). The use of propolis was not found in the development of composite resins in scientific literature and in technological literature.

The Brazilian red propolis enriched composite resin developed in this study showed antibacterial activity against *S. mutans* CCT 3440 after 1 h of direct contact and biocompatibility with 3T3 fibroblasts. Direct contact test has been used for determination of antibacterial and antifungal activity of enriched dental materials (Tavassoli Hojati et al., 2013; Zhang et al., 2017). The antibacterial activity of Brazilian red propolis enriched composite resins is a result of synergism of several markers present in BRP (Xie et al., 2014). Some examples of antimicrobial action mechanism for flavonoids presents in BRP are listed in the scientific literature as inhibition of nuclei acids synthesis (quercetin, chrysin, kaempferol), inhibition of quorum sensing (naringenin, kaempferol) and membrane disruption (naringenin, catechin) (Silva et al., 2016; Wang et al., 2018; Biharee et al., 2020).

In a study, the isolated flavanone naringenin had a MIC value of 500 μg/ml (1.84 mM) against *S. aureus*, a Gram positive bacteria (Wang et al., 2018). The same study showed that at 400 μg/ml (1.47 mM), naringenin down-regulated expression levels of *fabD, fabF, fabG, fabH and fabI*, genes associated with biosynthesis of fatty acid from cell membrane of *S. aureus*. The authors report that this down-regulating leading to modification of cell membrane fatty acid content, increasing the membrane fluidity and resulting in decrease of bacterial cell viability (Wang et al., 2018). Naringenin and other flavanones as liquiritigenin, 6-methoxyflavanone, 6-hydroxynaringenin, pinostrobin and pinocembrin was identified in EABRP used in this study. In the scientific literature, the structure activity relationship of flavanones was evaluated for the against Gram positive and was observed that two hydroxyl groups in the ring A at the C-5 and C-7 positions and none hydroxyl group in the ring B is a major contributing factor towards antibacterial activity, as pinocembrin (Echeverr et al., 2017). Studies of Chalcones structure activity relationship highlight the importance of hydroxyl group at C2′, C4′ and C4 for antibacterial activity (Ávila et al., 2008; Xie et al., 2014). The chalcone isoliquiritigenin, quantified in EABRP at concentration of 4.53 μg/ml, has this chemical arrangement.

New dental composites have been developed from the incorporation of antibacterial agents (Beyth et al., 2014). These agents can be inorganic particles, modified monomers or additives incorporated into both the polymer matrix and the charge particles, such as zinc oxide nanoparticles (Beyth et al., 2013, 2014; Rüttermann et al., 2013; Tavassoli Hojati et al., 2013; Inagaki et al., 2016), silica nanoparticles functionalized with amphotericin B (Lino et al., 2013), silver nanoparticles (Fan et al., 2011), modified monomers containing quaternary ammonium (Imazato et al., 2003; Makvandi et al., 2016, 2018) and antibacterial agents immobilized in composite (Namba et al., 2009; Mankovskaia et al., 2013). The concept of modified surfaces is reported in the scientific literature mainly for the purpose of developing antimicrobial and bioequivalent health materials (Busscher et al., 2012; Silva et al., 2016). Thus, surface-modified materials can be classified in five different ways: nonadhesive, tissue-integrating, contact-killing, antimicrobial-releasing coatings and multifunction coatings (Busscher et al., 2012).

The evidence that composite resins normally release some free monomers both in the oral cavity and in the dental pulp and that this release can cause toxicity is already well established in the literature (Schweikl et al., 2006; Nocca et al., 2011). In addition, there is a positive correlation between the toxicity of these composites and their monomers with oxidative stress that they can cause to normal cells in the oral cavity (Krifka et al., 2012, 2013; Perduns et al., 2019). The enzyme glutathione peroxidase (GPx1) it is present in both cell cytoplasm and mitochondrial matrix and acts reducing H_2_O_2_ to H_2_O when reduced glutathione (GSH), an tripeptide and endogenous antioxidant, levels are high (Krifka et al., 2012; Sies et al., 2017). The mechanism of oxidative stress in oral cell caused by methacrylate monomers involves the decrease in cellular levels of GSH due to the formation of the GSH-methacrylate monomer adduct that leads to glutathione peroxidase (GPx1) inhibition with a consequent increase in H_2_O_2_ levels in cell. Accumulation of H_2_O_2_ into oral cells leads to oxidative stress, in this process started by exposure to methacrylate monomers (Krifka et al., 2012). To reduce this GSH-methacrylate monomer adduct formation, studies have developed composite resins containing antioxidants species as ascorbic acid and N-acetylcysteine (Yang et al., 2019). The Brazilian red propolis DPPH radical scavenging capacity suggest a possible explanation for the statistical difference found between enriched and commercial composite resin in the cytotoxicity assay in this study, when the phenolic compounds of BRP can be inhibition the GSH-methacrylate monomer. More studies are necessary to confirm this supposition.

The enriched Brazilian red propolis composite resin exhibited flexural resistance higher than 100 MPa, exceeding the 80 MPa values indicated for polymer-based restorative materials (International Organization for Standarization, 2000), In the mechanical tests of flexural resistance and Knoop microhardness, the insertion of the HEMA diluent was determinant for the reduction of the properties observed between the RC group and the other groups. The DC% of 60% for filtek bulk fill flow composite resin is according with scientific literature data (Pongprueksa et al., 2015). The increase of DG% exhibited by modified composite resin (RS and RPs) was provided by EABRP-HEMA incorporation. This fact is confirmed by thermal analysis data, where RP15 and RP25 showed increase in thermal resistance when compared to RC. This increase on thermal resistance happens by EABRP-HEMA addition that promoted formation of cross-links in the 3D structure of the polymer (Achilias et al., 2010; Vouvoudi et al., 2015). The main monomer at the RPs composition, the urethane dimethacrylate, is able to form hydrogen bonds with the phenolic compounds present in the red propolis, making the polymeric network more cross-linked and consequently more thermal resistant (Achilias et al., 2008). The bigger DC% for RP25 in comparation with DC% for RC mean a smaller amount of free monomers in enriched composites that contributes for the smaller cytotoxicity exhibited by RP25.

Sorption has an important negative correction with the amount of composite filler. When the percentage filler increases, the polymer matrix decreases and consequently decreases water sorption, which is a phenomenon associated mainly with the polymer matrix (Alshali et al., 2015). In this study, all BRP enriched resins showed a significant increase in water sorption. Even so, the values were lower than the maximum recommended value of 40 μg/mm^3^ according to the ISO standard for restorative resins (International Organization for Standarization, 2000). Finally, the roughness of composite resins can be classify as mild (Ra ≤ 25 nm), moderately rough (25 mn > Ra ≤ 150 nm) and rough (>150 nm) (Mei et al., 2011). According to this classification, all composites tested RC, RS, RP10, RP15 and RP25 are considered as mildly roughness.

## Conclusion

Up to our knowledge, this is a first study which describes the use of propolis on composite resins development. The phytochemical profile of Brazilian red propolis was characterized and used BRP extract showed up bacteriostatic and bactericide against *Streptococcus mutans*. A BRP enriched composite resin (RP25) was obtained by commercial composite resin modification. RP25 exhibited antibacterial activity against *S. mutans* by direct contact test and no showed cytotoxicity against 3T3 fibroblasts. The RP25 exhibited compatible mechanical and physical-chemical properties to the indicate for composite resins. Finally, more one application of propolis on development of materials for healthcare was carried, obtaining a BRP enriched composite resin characterized as an unprecedented, biocompatible, and antibacterial dental material.

## Data Availability

The raw data supporting the conclusions of this article will be made available by the corresponding author on request.
