# Supplementary material for: Propolis in Oral Healthcare: Antibacterial Activity of a Composite Resin Enriched With Brazilian Red Propolis
Source: Front Pharmacol. 2021 Nov 29;12:787633. doi: 10.3389/fphar.2021.787633 (PMC8667603; doi:10.3389/fphar.2021.787633)
Supplement: Supplementary file 1 [file Image2.pdf]

Supplementary Material

Supplementary Figure

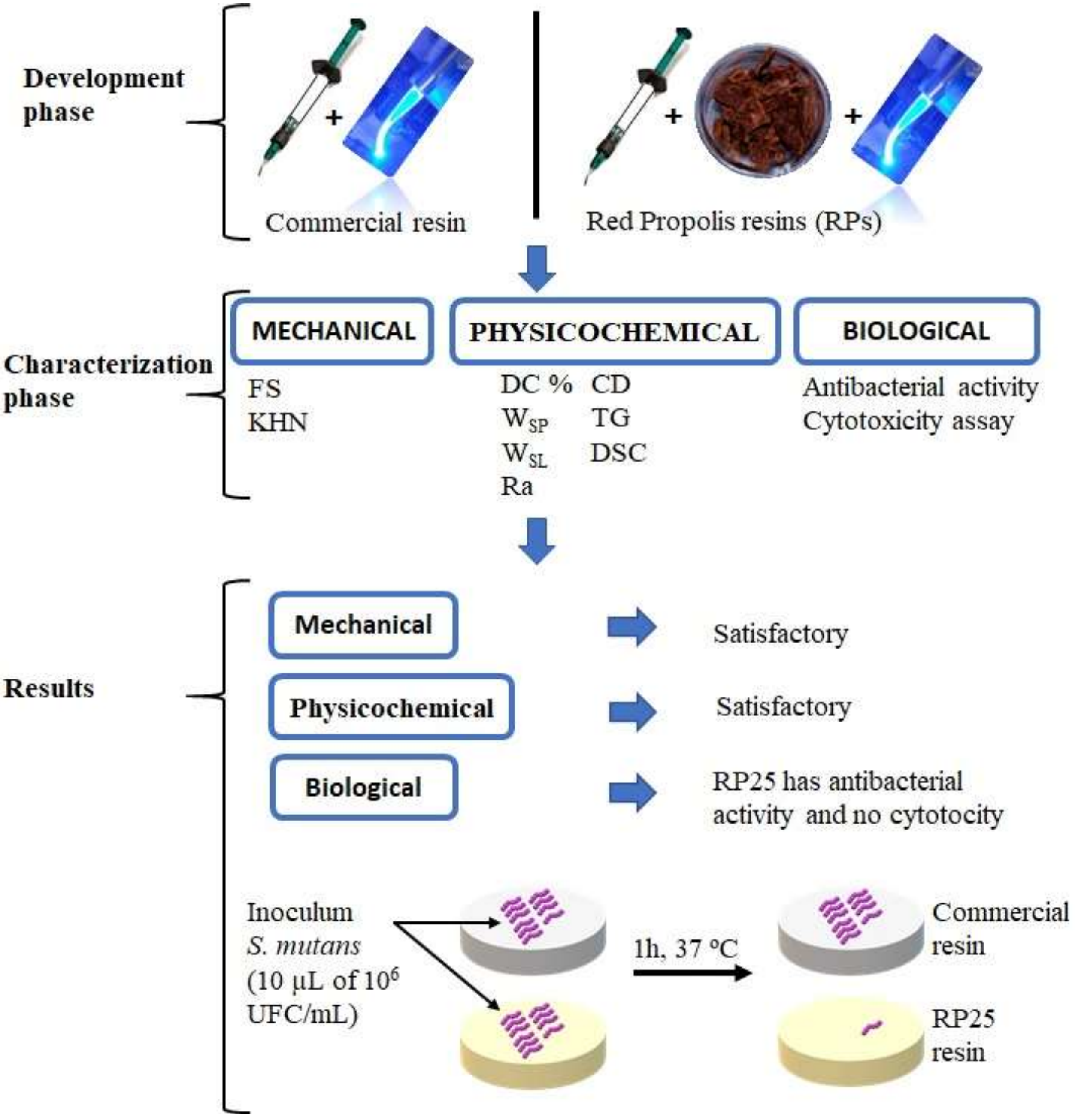

Supplementary Figure 2. Schematic representation of the process of obtaining and characterization of composite resin enriched with Brazilian red propolis.
