# Supplementary material for: Propolis in Oral Healthcare: Antibacterial Activity of a Composite Resin Enriched With Brazilian Red Propolis
Source: Front Pharmacol. 2021 Nov 29;12:787633. doi: 10.3389/fphar.2021.787633 (PMC8667603; doi:10.3389/fphar.2021.787633)
Supplement: Supplementary file 2 [file Image1.pdf]

Supplementary Material

Supplementary Figure

Phenolic acids

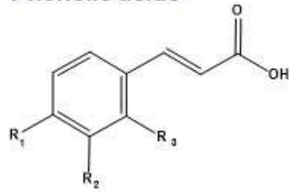

	R_1	R_2	R_3
**1**	OH	OH	H
**2**	OH	OCH_3	H
**3**	OH	H	OH
**4**	OH	H	H

Flavones

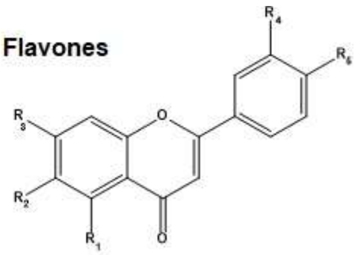

	R_1	R_2	R_3	R_4	R_5
**6**	OH	OCH_3	7-rhamnoside	OH	OH
**32**	OCH_3	H	OH	H	OH
**34**	OH	H	OH	H	H

Isoflavones

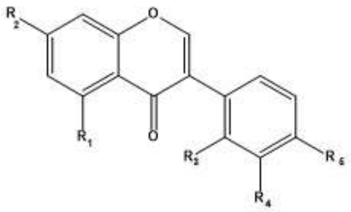

	R_1	R_2	R_3	R_4	R_5
**5**	OCH_3	O-beta-glucopyranosyl	H	H	OH
**7**	OH	OH	H	H	OH
**10**	OH	OH	OH	H	OH
**12**	H	OH	H	H	OH
**14**	H	OH	OH	H	OCH_3
**18**	H	OH	H	OH	OCH_3
**22**	H	OH	H	H	OCH_3
**23**	H	OCH_3	H	H	OH
**33**	OH	OH	H	H	OCH_3

Flavanonols

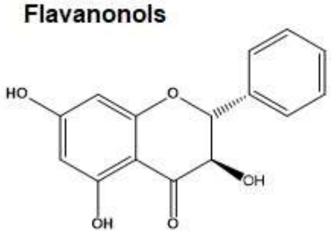

17 Pinobanksin

Flavonols

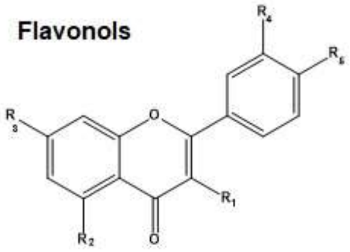

	R_1	R_2	R_3	R_4	R_5
**8**	OH	OH	OH	H	OH
**36**	OCH_3	H	O-(2-hydroxy-ethyl)	O-benzyl	O-benzyl

Flavan

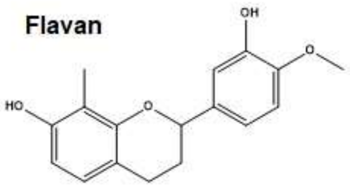

39 7,3'-dihydroxy-4'-methoxy-8-methylflavane

Flavanols

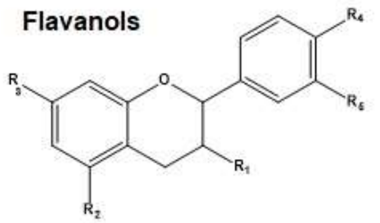

	R_1	R_2	R_3	R_4	R_5
**9**	(+) OH	OH	OH	OH	OH
**11**	(-) OH	OH	OH	OH	OH

C30 isoflavones

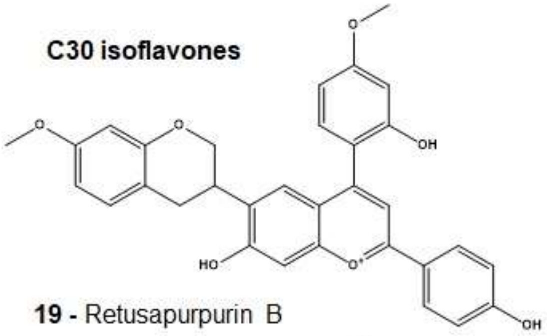

19 - Retusapurpurin B

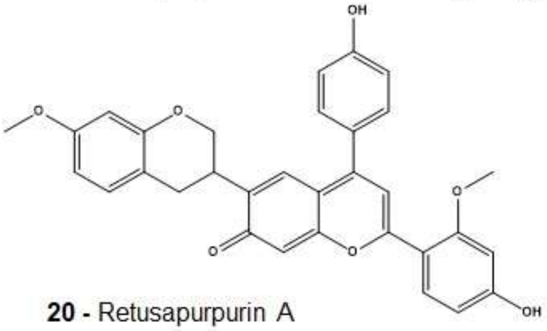

20 - Retusapurpurin A

Pterocarpan

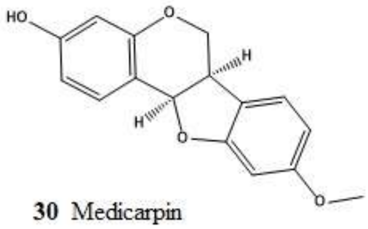

30 Medicarpin

Flavanones

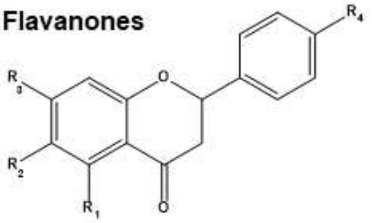

	R_1	R_2	R_3	R_4
**13**	H	H	OH	OH
**16**	OH	H	OH	OH
**24**	H	OCH_3	H	H
**25**	OH	OH	OH	OH
**29**	OH	H	OCH_3	H
**35**	OH	H	OH	H

Chalcones

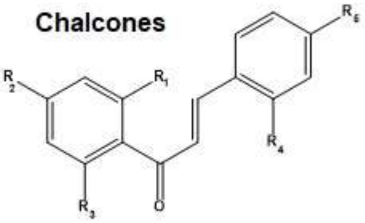

	R_1	R_2	R_3	R_4	R_5
**21**	OH	OH	H	H	OH
**26**	H	OH	H	OCH_3	OH

Dihydrochalcone

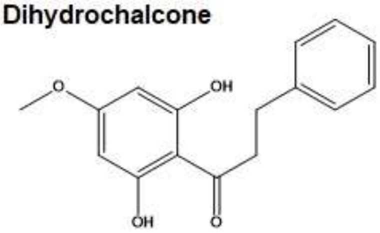

31 2',6'-dihydroxy-4'-methoxydihydrochalcone

Prenylated Benzophenones

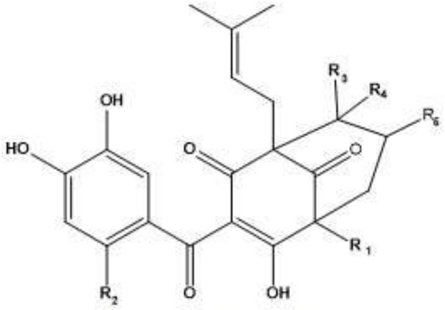

	R1	R2	R3	R4	R5
**42**	Prenyl group	OH	Prenyl group	CH_3	Prenyl group
**44**	Geranyl group	H	CH_3	CH_3	Prenyl group
**45**	Geranyl group	H	CH_3	CH_3	Prenyl group
**48**	Geranyl group	H	CH_3	Prenyl group	Prenyl group
**49**	Geranyl group	H	CH_3	Prenyl group	Prenyl group
**50**	Geranyl group	H	CH_3	CH_3	Geranyl group

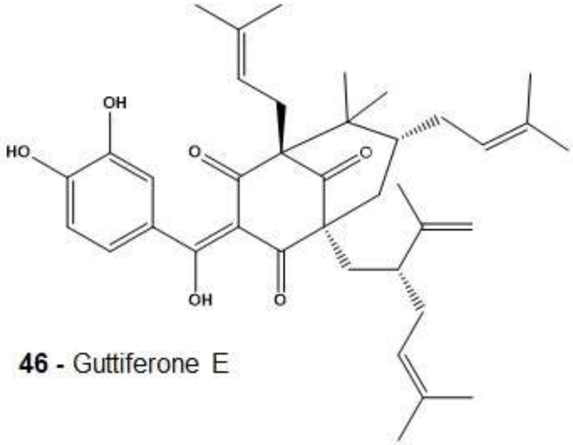

46 - Guttiferone E

Isoflavans

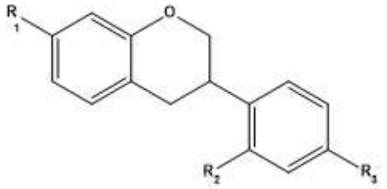

	R1	R2	R3
**28**	OH	OH	OCH_3
**37**	OH	OCH_3	OCH_3
**38**	OCH_3	OH	OCH_3

Triterpenes

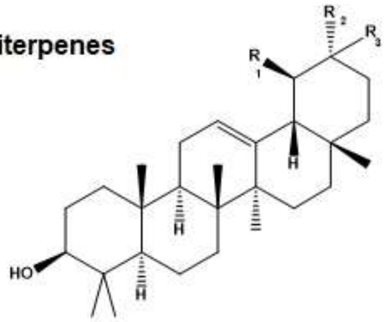

	R1	R2	R3
**40b**	CH_3	CH_3	H
**40c**	H,H	CH_3	CH_3

Supplementary Figure 1. Chemical structures of some of compounds identified from EABRP by LC-ESI-Orbitrap-FTMS.
